# The last 1100 years of activity of La Fossa caldera, Vulcano Island (Italy): new insights into stratigraphy, chronology, and landscape evolution

**DOI:** 10.1007/s00445-024-01738-4

**Published:** 2024-04-20

**Authors:** Federico Di Traglia, Marco Pistolesi, Costanza Bonadonna, Mauro Rosi

**Affiliations:** 1 Istituto Nazionale Di Geofisica E Vulcanologia, Osservatorio Vesuviano, Via Diocleziano 328, 80124 Napoli, Italy; 2 Department of Earth Sciences, University of Pisa, Via S. Maria 53, 56126 Pisa, Italy; 3 Department of Earth Sciences, University of Geneva, Rue Des Maraîchers 13, 1205 Geneva, Switzerland

**Keywords:** Volcano stratigraphy, Active caldera, Tephra, Historical eruptions, Island of Vulcano, Aeolian archipelago

## Abstract

A detailed study of past eruptive activity is crucial to understanding volcanic systems and associated hazards. We present a meticulous stratigraphic analysis, a comprehensive chronological reconstruction, thorough tephra mapping, and a detailed analysis of the interplay between primary and secondary volcanic processes of the post-900 AD activity of La Fossa caldera, including the two main systems of La Fossa volcano and Vulcanello cones (Vulcano Island, Italy). Our analyses demonstrate how the recent volcanic activity of La Fossa caldera is primarily characterized by effusive and Strombolian activity and Vulcanian eruptions, combined with sporadic sub-Plinian events and both impulsive and long-lasting phreatic explosions, all of which have the capacity to severely impact the entire northern sector of Vulcano island. We document a total of 30 eruptions, 25 from the La Fossa volcano and 5 from Vulcanello cones, consisting of ash to lapilli deposits and fields of ballistic bombs and blocks. Volcanic activity alternated with significant erosional phases and volcaniclastic re-sedimentation. Large-scale secondary erosion processes occur in response to the widespread deposition of fine-grained ash blankets, both onto the active cone of La Fossa and the watersheds conveying their waters into the La Fossa caldera. The continuous increase in ground height above sea level, particularly in the western sector of the caldera depression where key infrastructure is situated, is primarily attributed to long-term alluvial processes. We demonstrate how a specific methodological approach is key to the characterization and hazard assessment of low-to-high intensity volcanic activity, where tephra is emitted over long time periods and is intercalated with phases of erosion and re-sedimentation.

## Introduction

Constraining the chronology and eruptive styles of past volcanic activity is fundamental both for accurate hazard assessments and for understanding unrest signals recorded by monitoring networks (Acocella et al. 2015; Rosi et al. 2022). Detailed investigations of eruptive histories have traditionally relied on thorough reconstructions of tephro-chronological frameworks based on lapilli fallout beds resulting from large-volume, sustained explosive volcanic eruptions (Crandell et al. 1975; Sheridan et al. 1981; Thorarinsson 1981; Pistolesi et al. 2011; Nakamura 2016; Freundt et al. 2023). Robust lateral tracing of lapilli beds at continental central volcanoes has played a crucial role in the accurate reconstruction of their past eruptive activity. These eruptions result in the emplacement of continuous tephra blankets, each with well-recognizable features, that are resistant to erosion, and which allow for correlation over regional areas and that can be coupled with distal ash beds deposited in marine or lacustrine settings (Albert et al. 2017; Tomlinson et al. 2015).

In contrast, volcanoes prone to low- to moderate-intensity explosive activity, such as phreatic, Surtseyan, Strombolian, violent Strombolian, and Vulcanian eruptions, are characterized by the repetitive emplacement of fine-grained and moderately dispersed tephra beds in the down-wind sectors from the source vent, resulting in more complex tephro-stratigraphic studies (Walker 1973; Newhall and Self 1982; Arrighi et al. 2001; Yasui and Koyaguchi 2004; de Vita et al. 2010; Pistolesi et al. 2016, 2017; Bernard and Bouvet de Maisonneuve 2020; Belousov et al. 2021; Drymoni et al. 2023; Vallejo et al. 2024).

**Fig. 1:**
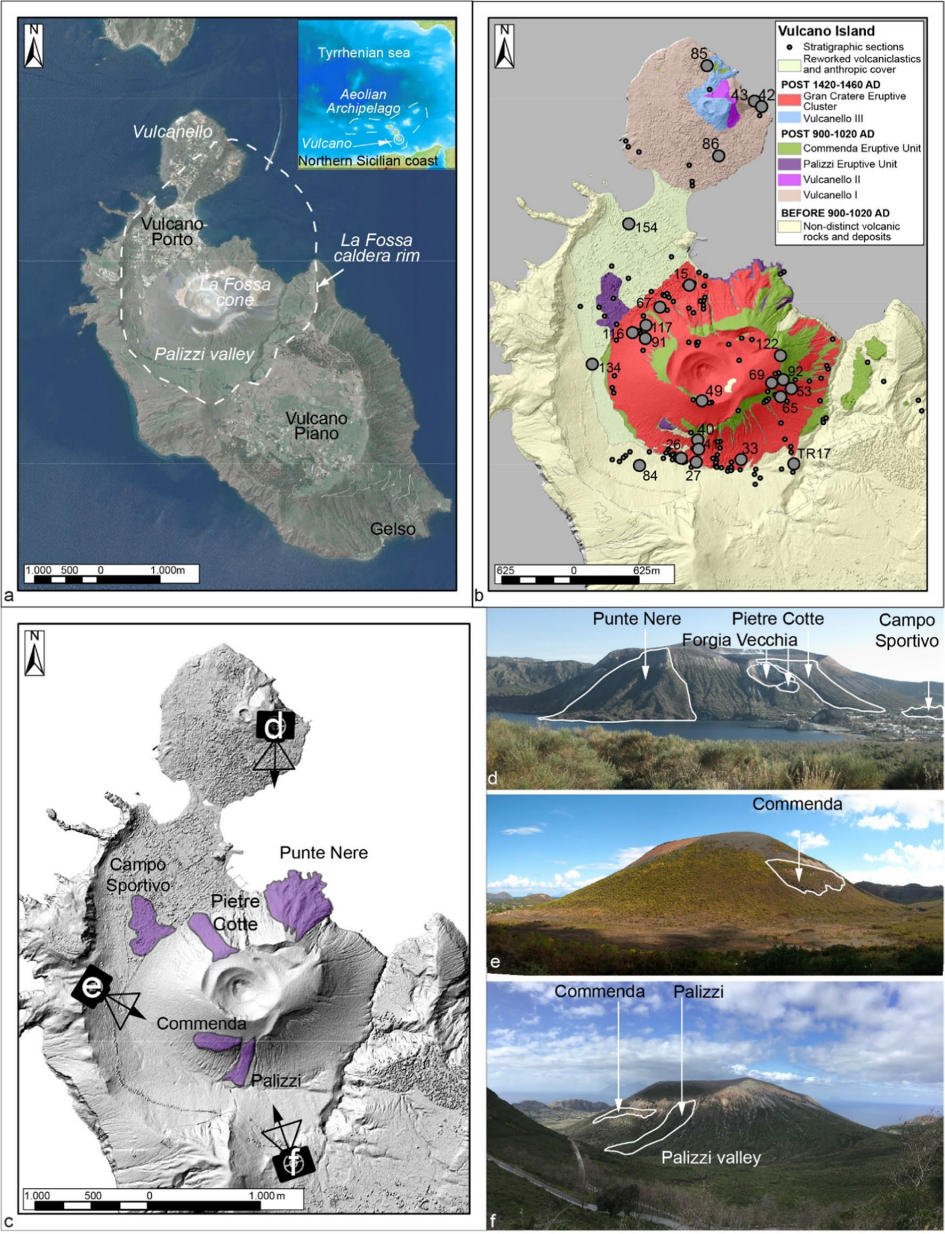
**a** Geographical overview of the island of Vulcano, with the main features highlighted. The caldera boundaries are modified after Giocanda and Sbrana (1991), Romagnoli et al. (2012) and Casalbore et al. (2019); **b** simplified geological map of the northern area of the island (the stratigraphic units are in chronological order), modified after Di Traglia et al. (2013) and Fusillo et al. (2015). **b** Location of the stratigraphic sections analyzed in this work. The sections that are cited in this work have been highlighted. **c** Area occupied by the post-900 AD lava flows emitted by the La Fossa cone and location of photos **d**, **e**, and **f**: **d** Punte Nere, Pietre Cotte, and Campo Sportivo lava flows photographed from Vulcanello. It is also possible to observe the location of the eccentric craters of the Forgia Vecchia. **e** Commenda lava, photographed from the Lentia domes. **f** Palizzi and Commenda lava flows photographed from the northern limit of the Piano area. The initial part of the Palizzi valley can be observed

The recent (AD 900–1890) activity of the La Fossa Caldera on the Island of Vulcano, located in the Aeolian archipelago in the Southern Tyrrhenian Sea (Italy) (Fig. 1), presents many of the difficulties listed above. Its eruptive activity includes a high number of eruptive episodes of variable magnitude, style, and duration, from several vents located within a caldera system. The most recent activity in 1888–90 represents the prototype of Vulcanian eruption (Mercalli and Silvestri. 1891; De Astis et al. 2013; Di Traglia et al. 2013; Fusillo et al. 2015; Rosi et al. 2018; Malaguti et al. 2022). Since then, the caldera, which hosts an important hydrothermal system (Fulignati et al. 1998), has experienced several periods of unrest, the last one starting in August–September 2021 and still ongoing at the time of writing (September 2023), which culminated in a partial evacuation of the island because of strong degassing increase in inhabited areas (Aiuppa et al. 2022; Di Traglia et al. 2023). All these factors, combined with the remarkable vulnerability of the island due to uncontrolled urban development over the past 70 years and a significant seasonal variation of the exposed population, result in a high volcanic risk (Galderisi et al. 2013; Biass et al. 2016a, b; Bonadonna et al. 2022; Bonasia et al. 2022). For these two main reasons (high risk and state of unrest), an improved assessment of the eruptive history and volcanic hazards has become fundamental and timely. Overall, this study consists of a detailed analysis of the tephro-stratigraphic record which explores with high precision the intricacies of activity between the fifteenth and nineteenth centuries. In this paper, we revise previous stratigraphic analyses and provide a more accurate chrono-stratigraphic framework of the recent eruptive systems (La Fossa cone and Vulcanello) based on a better characterization of the chronology of volcanic events, a more accurate classification of the various eruptive styles and a detailed characterization of emplacement and depositional processes of the products. Age attribution to the identified tephra deposits was made possible by a revision of the historical reconstruction of the events combined with tephra characteristics, which represents the first of its kind for the island (Di Traglia et al. 2013; Fusillo et al. 2015; Selva et al. 2020; Malaguti et al. 2022). In addition, we use La Fossa caldera as an example to describe a comprehensive approach for the characterization of volcanic systems associated with low-to-high intensity, long-lasting activity intercalated with significant erosional phases.

## Geological setting

Vulcano is famous for giving its name to one of the most common style of explosive volcanic activity. The eruption in 1888–1890 was described in detail in a milestone work by Mercalli and Silvestri (1891) and taken as a reference for the Vulcanian style (Walker 1973). Given its continuous state of activity, since Greek and Roman times the island was considered the home of fire gods (for the Greeks' Ιερα and for the Romans Hierà, Therasia, and Vulcania).

Vulcano is one of the volcanic islands and seamounts forming a ring-shaped volcanic structure located in the Southern Tyrrhenian Sea (Favalli et al. 2005; Fig. 1a). As part of the southern Aeolian Islands, Vulcano is crossed by the regional NNW–SSE tectonic trend (Barreca et al. 2014), and a N-S alignments of recent vents (Ruch et al. 2016). The volcanic structure includes two calderas, an older one to the south (Il Piano) and a younger one to the north, the 4 × 2 km La Fossa Caldera, whose origin is still debated (Gioncada and Sbrana 1991; Ventura et al. 1999; Romagnoli et al. 2012; De Astis et al. 2013; Ruch et al. 2016; Casalbore et al. 2019).

The magmatic feeding system beneath Vulcano Island is considered polybaric and characterized by magma storage zones that have persisted throughout the last 120 ka from about 20 km to 5–3 km of depth, and a shallow storage zone at 1–2 km beneath the La Fossa cone (Clocchiatti et al. 1994a, b; Gioncada et al. 1998; Zanon et al. 2003; Chiarabba et al. 2004; Fusillo et al. 2015; Nicotra et al. 2018; Alparone et al. 2019; Costa et al. 2020, 2023; Aiuppa et al. 2022).

After the formation of the caldera, recent volcanic activity focussed at two centres: La Fossa cone, a composite volcano located in a central-southern position with respect to La Fossa Caldera, and Vulcanello, a small composite volcano located along the northernmost border of the caldera (Fig. 1a). The composition of the magmas erupted at Vulcano varies from latite to rhyolite, with minor shoshonites (Keller 1980; De Astis et al. 1997; Gioncada et al. 2003; Fulignati et al. 2018; Costa et al. 2020). The stratigraphic record of recent volcaniclastic deposits (since ~ 900 AD; Malaguti et al. 2022) of the La Fossa cone has been subdivided into two main stratigraphic clusters (Di Traglia et al. 2013), namely the Palizzi-Commenda Eruptive cluster (PCEC) (further subdivided into the Palizzi Eruptive Unit (PEU) and the Commenda Eruptive Unit (CEU)), and the Gran Cratere eruptive cluster (GCEC).

The post-900 AD eruptive activity includes Vulcanian eruptions, sub-Plinian eruptions, phreatic explosions, and minor lava effusions of both trachytic and rhyolitic composition (Fig. 1c, d, e, f; Di Traglia et al. 2013; Selva et al. 2020). The phreatic activity includes the violent eruption of the Breccia di Commenda between the end of the thirteenth and the beginning of the fourteenth century AD (Rosi et al. 2018), as well as the two eccentric eruptions in the northern flank of La Fossa cone (“Forgia Vecchia” area; Sheridan and Malin 1983).

Vulcanello is a small peninsula comprising a 123-m-high composite edifice formed by three, partially overlapping, scoria cones aligned NE–SW along the northern ring fault of La Fossa caldera, and a lava platform (Davì et al. 2009; De Astis et al. 2013; Romagnoli et al. 2012; Fusillo et al. 2015). Early eruptions of Vulcanello likely occurred in 126 or 183 BC, as reported by the Strabo and Plinius historical chronicles quoted by Mercalli and Silvestri (1891) and De Fiore (1922). However, recent studies by Arrighi et al. (2006), Fusillo et al. (2015), Malaguti et al. (2022), and Manni and Rosi (2021) revealed that the islet definitively emerged from the ocean during Middle Ages likely on the shallow seabed left by the previous eruptions occurred in Roman time. The early Medieval activity (AD 899–1044; Malaguti et al. 2022) was responsible for the build-up of the main part of the peninsula, with the growth of two cones associated with two lava fields (the Vulcanello lava platform and a submarine pillow lava field). The second cluster of eruptions built the third cone, associated with the Roveto (AD 1420–1460; Malaguti et al. 2022) and Valle dei Mostri lava flows (AD 1400–1466 or 0.397 ± 0.097 ka; Malaguti et al. 2022 and Keller 1980, respectively). Vulcanello concluded its activity in the sixteenth century, and the following fumarolic activity ended few decades ago.

## Methods

In this study, the following nomenclature for clastic deposits resulting from eruptive activity, geomorphological processes, and sedimentary processes in volcanic areas is employed:

- the term “tephra” is applied to pyroclastic material that is ejected during volcanic explosions and travels through the atmosphere before depositing on the ground (Thorarinsson 1981);
- “volcaniclastic deposit” refers to clastic deposits whose particle composition originates from volcanic sources. Within this category, two sub-groups are used following the classification proposed by Di Capua et al. (2023 and references therein):
- “primary volcaniclastic deposits” are directly generated by volcanic activity, with the deposit formation mechanism being intrinsically linked to volcanic processes, encompassing both generation and transport/deposition mechanisms;
- “secondary volcaniclastic deposits” denote reworked/resedimented deposits comprising clasts of volcanic origin. In such instances, sediments have been mobilized and deposited from an unconsolidated surface source through non-volcanic processes.

**Fig. 2:**
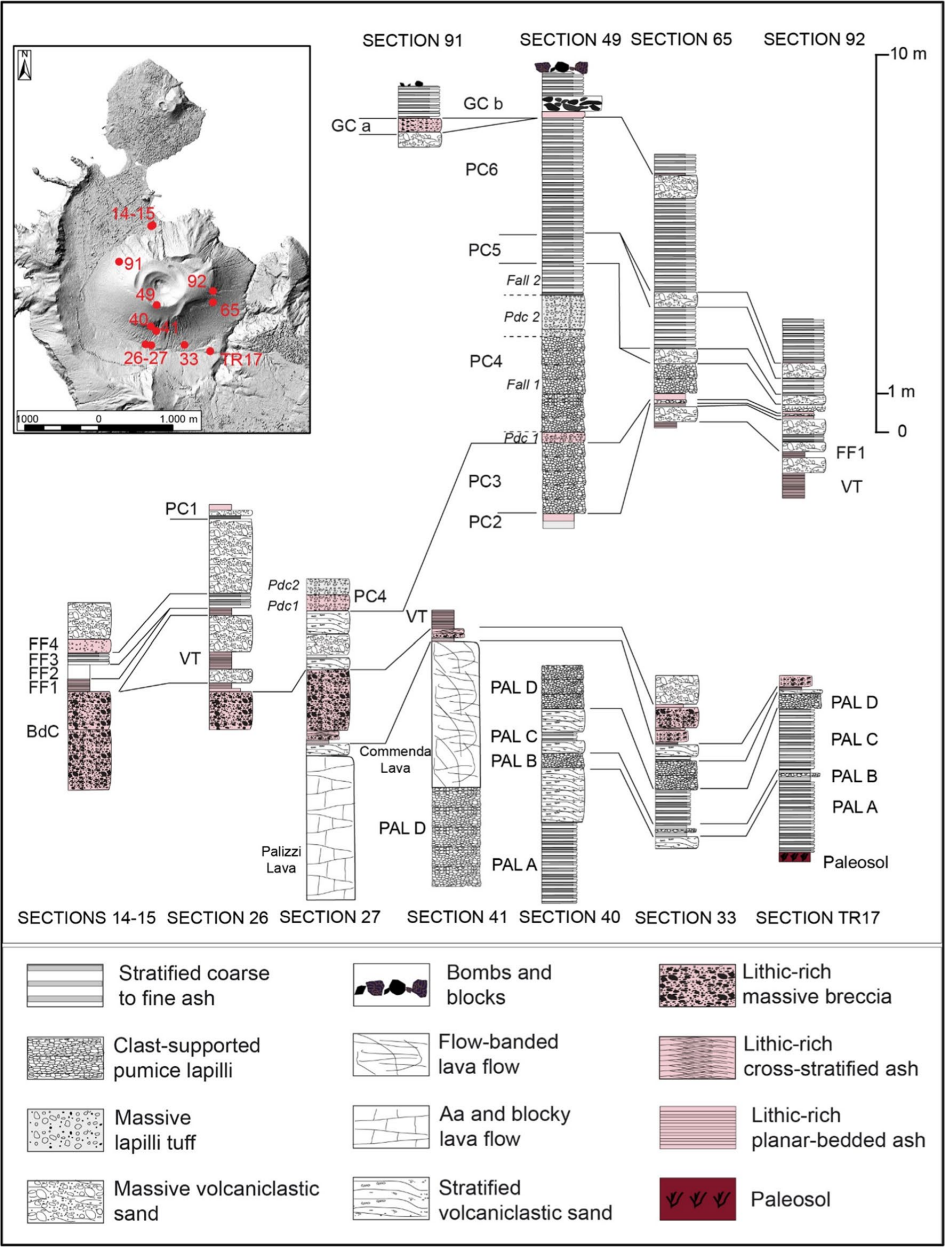
Location of the main sections analyzed for stratigraphic correlations. They are all natural outcrops except for TR17, which is a mechanically excavated trench

The stratigraphic work consisted of the analysis of > 150 stratigraphic sections, mainly located within the La Fossa caldera (Figs. 1b, 2) and carried out over the last 15 years. In addition to more than 130 natural cuts, ~ 20 trenches (1 to 7 m deep) were mechanically and hand excavated in sites deemed favorable for the accumulation of complete tephra sequences to better understand the stratigraphy and to improve the mapping of tephra layers. The analysis focused on the following: (i) the identification of erosive unconformities and/or interposition of secondary deposits indicating periods of stasis and/or change of eruptive activity (labeled from EU1 to EU7); (ii) the description of tephra bed characteristics (internal structure, overall granulometry, bedding characteristics, and nature of volcanic components); (iii) the systematic collection at different sites of complementary sedimentological features of each bed (e.g., deposit thickness for drawing of isopach maps, maximum clast size, depositional facies). Field observations also allowed the reconstruction of the morphological-stratigraphic evolution of the La Fossa Caldera bottom where the present main village is located.

The chronological attribution of deposits to eruptive events described in the historical record was accomplished by comparing the characteristics and of individual tephra packages, with information provided by the chronicles (age, duration, sectors where volcanic materials were dispersed and particle size of ejected materials). The areal distribution of specific tephra layers was used to support the correlation between stratigraphic data and historical accounts. The historical records primarily stem from the pioneering studies conducted by Mercalli and Silvestri (1891) and De Fiore (1922). These works not only offer stratigraphic and descriptive information on the eruptive and fumarolic activity of Vulcano island during the late nineteenth and early twentieth centuries, including a comprehensive account of the last eruption (1888–1890), but also provide a compilation of bibliographic references. These sources also contain fundamental discussions regarding the reliability of the referenced materials. Finally, the work took great advantage with the recent geochronological and tephrochronological works (Arrighi et al. 2006; Pistolesi et al. 2021; Malaguti et al. 2022) as they all concur in providing a solid chronological base on the volcanic history of the time period 900–1400 AD.

## Results

### Analysis of the stratigraphic sequences

**Fig. 3:**
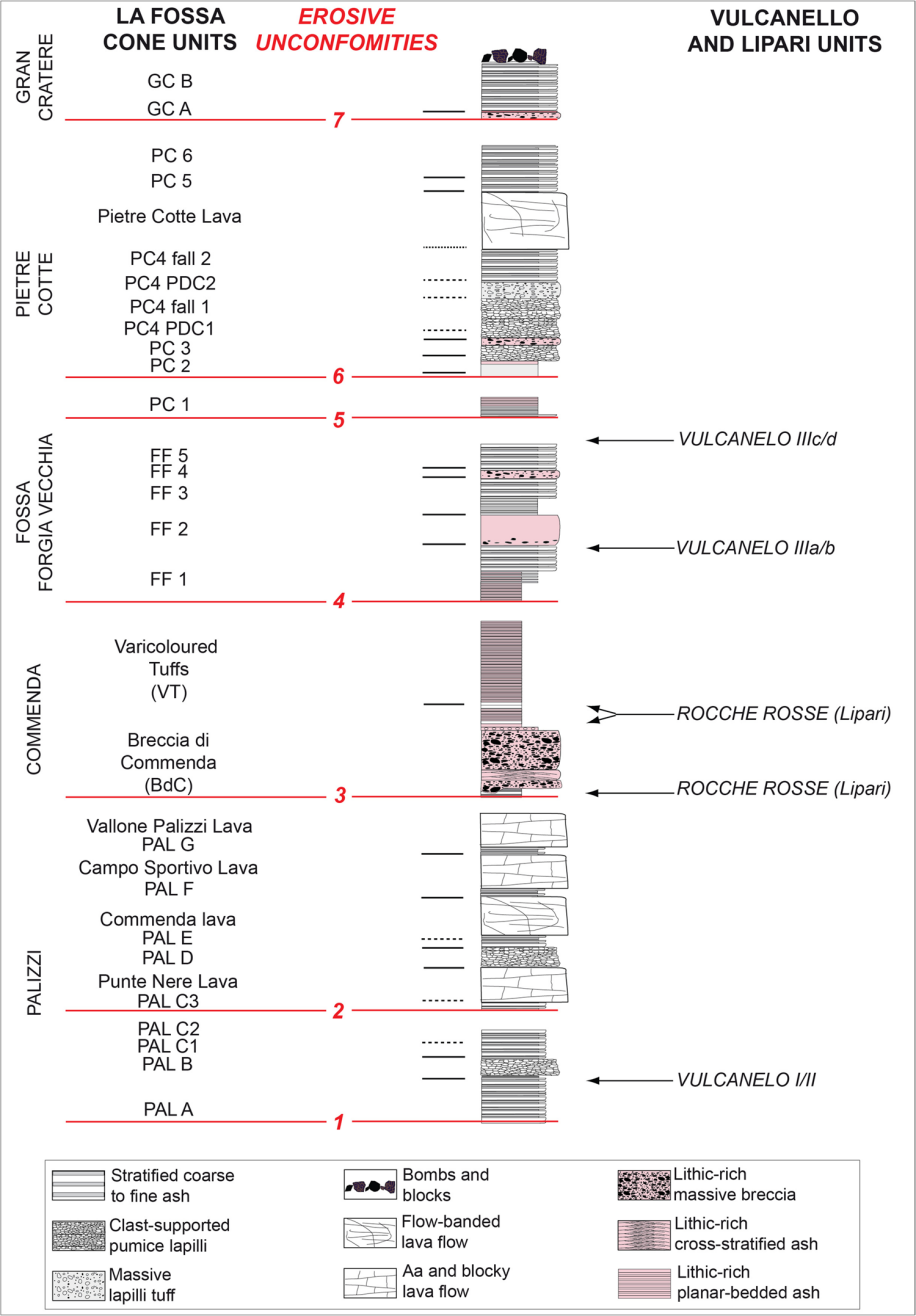
Stratigraphic scheme of the post-900 AD activity of the La Fossa cone, also including main unconformities (EU1 to EU7, red lines) recognized throughout the volcano-sedimentary sequence

The overall tephra framework since ~ 900 AD (i.e., from the Palizzi sequence (PEU) to nineteenth century (1888–1890)), is introduced in Figs. 2 and 3. For the purpose of the work, tephra can be conveniently referred to different categories: (i) stratified, coarse to fine ash deposits, (ii) clast-supported, pumice lapilli beds, (iii) massive lapilli (pumices and/or dense clasts) tuff, (iv) bombs and block deposits, (v) lithic-rich, massive breccia, (vi) lithic-rich, cross stratified ash beds, (vii) lithic-rich, planar-bedded ash beds. In addition, some lava flow units (flow-banded or aa and blocky lava flows) are intercalated with the tephra sequences. Massive to stratified, secondary volcaniclastic deposits (Di Capua et al. 2023) are also interbedded to tephra in the topographic lows, reflecting eruptive stasis during which fine-grained tephra was mobilized by exogenous activity. In Figs. 2 and 3, different tephra categories are indicated with different colors and symbols whereas the different tephra units are labeled and correlated using the most complete sections.

### The Palizzi (PEU) sequence

**Fig. 4:**
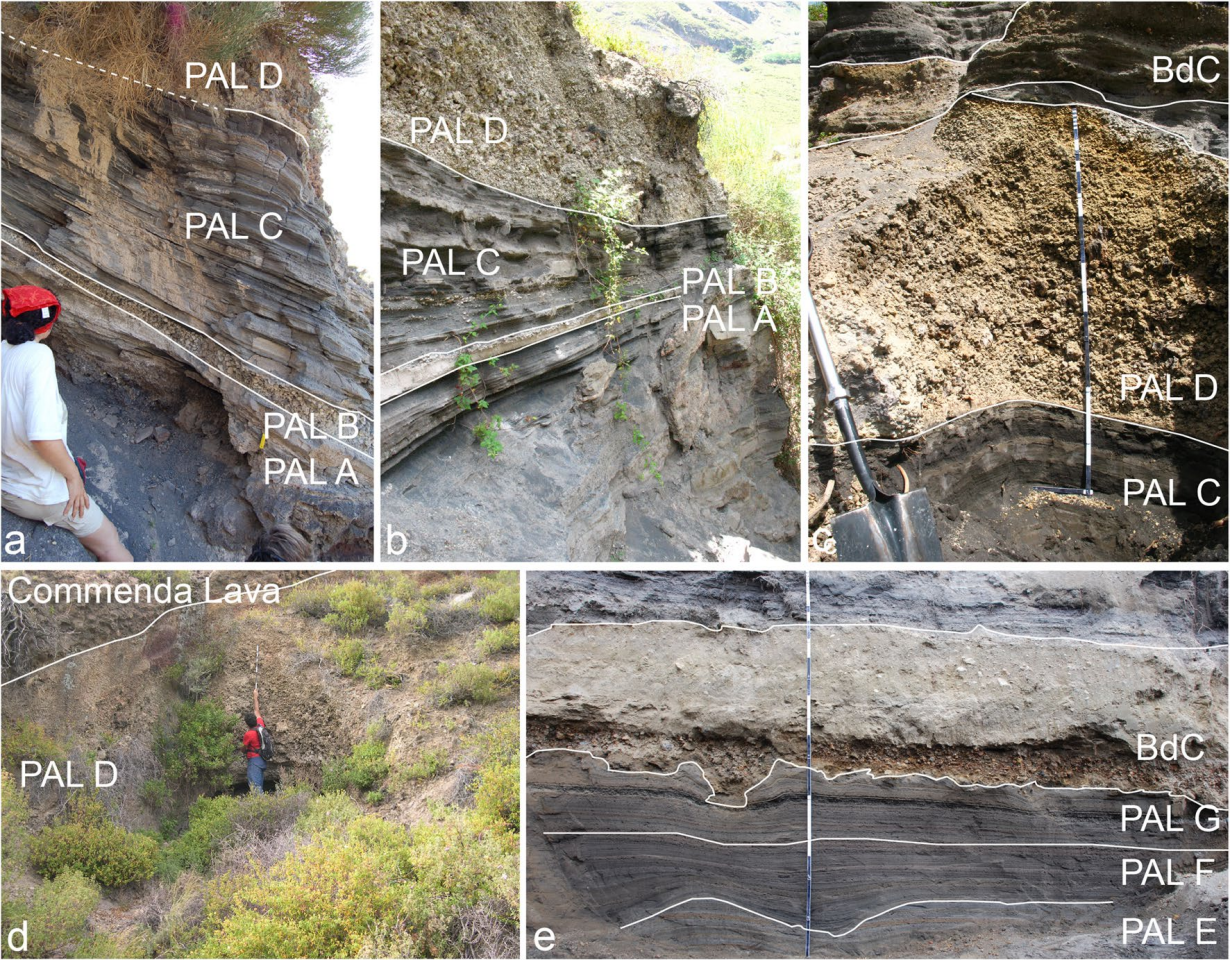
Main stratigraphic sections showing the tephra beds associated with PEU. **a** Sequence from PAL A to PAL D outcropping in Sects. 41 (Figs. 1 and 2), **b** evidence of re-sedimentation of volcaniclastic material deriving from the erosion of PAL A and PAL B, **c** details of PAL D deposit where the maximum thickness is observed: the tephra is directly overlain by the Commenda lava flow. **d** PAL E, PAL F, PAL G, and BdC in TR17 (see Figs. 1 and 2)

**Fig. 5:**
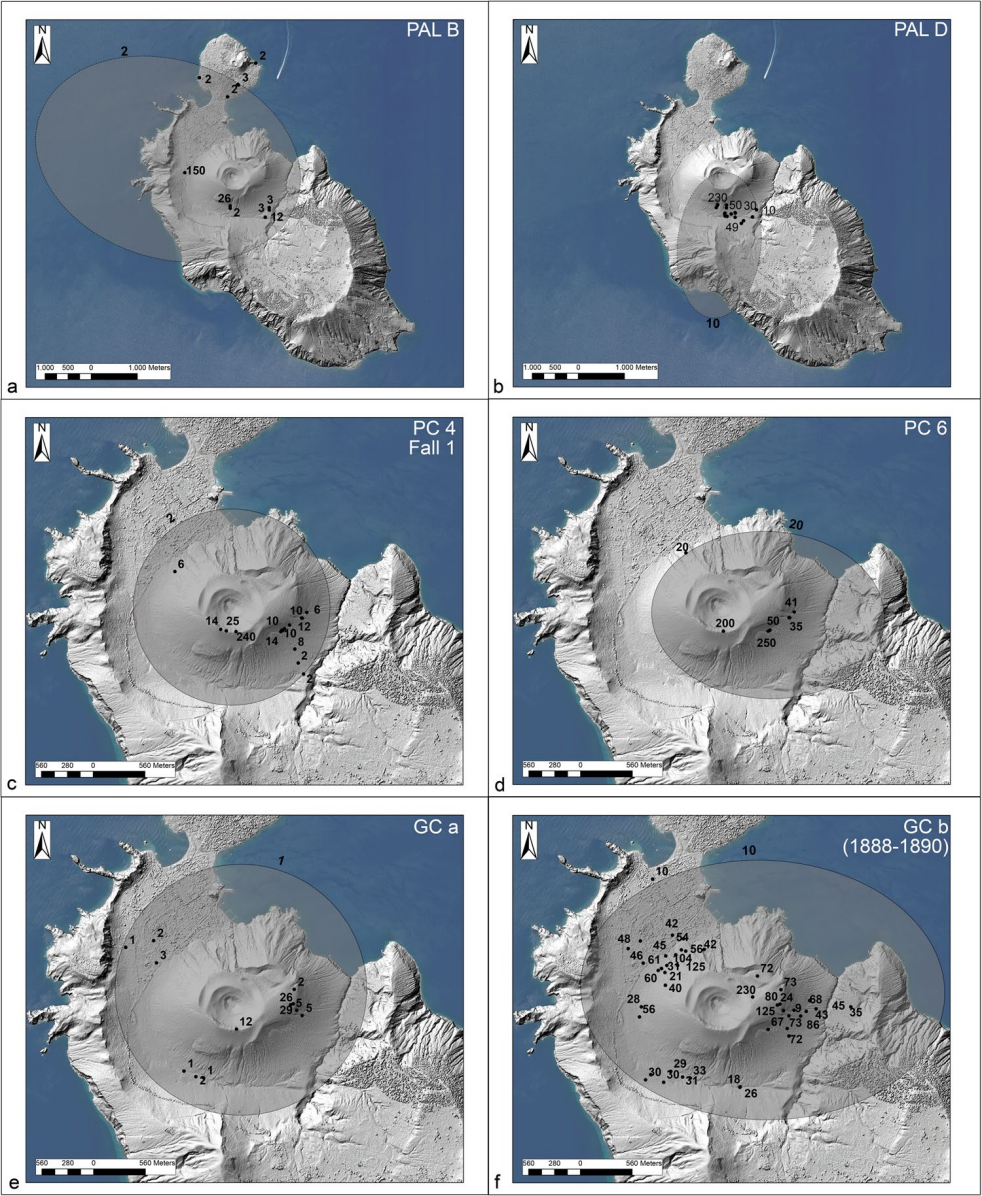
Areal distribution of the tephra fallout deposits (thickness values in mm) **a** PAL B; **b** PAL D; **c** PC 4 (Fall 1); **d** P C 6; **e** GCa, **f** GCb (the 1888–1890 Vulcanian eruption)

The Palizzi (PEU) eruptive unit (10th–late thirteenth century), produced by the La Fossa cone, consists of a series of dark-colored to black, parallel-bedded, ash layers punctuated by two coarse-grained lapilli beds (Fig. 3). The base-to-top tephra sequence was investigated in detail at a machine-excavated trench key section (TR17 in Fig. 2). This site provides a complete succession of eruptive materials accumulated on a flat area downwind of the vent and on a relative topographic high. The sequence rests on a humified horizon containing scattered, mm-seized charcoals fragments defined as unconformity EU1, corresponding to the S1 of Di Traglia et al. 2013 (Figs. 2 and 3) and recently dated at AD 900–1020 (Malaguti et al. 2022). The sequence (Fig. 4) can be conveniently split into 7 main sub-units (PAL A to PAL G, with PAL C further divided in PAL C1, PAL C2, and PAL C3; see also Costa et al. 2023) attaining a maximum thickness of ~ 5 m. PAL A consists of ~ 1 m-thick succession of dark grey to black, parallel-layered ash containing vesicular, fluidal glassy pyroclasts. It overlies a > 130 cm-thick, fine- to-coarse-grained loose, weakly weathered, massive ash older than the studied interval. PAL B is a multi-layered sequence with 2 cm of grey to white coarse ash to white-colored pumice lapilli at the base, overlain by 4 cm of grey-colored, fine-grained ash layers. Three cm of white, reversely to normally graded, coarse ash to white, pumice lapilli follow, representing the coarser and widely dispersed layer of PAL B tephra package. This lapilli-supported bed represents a marker unit across the northern sector of the island (Frazzetta et al. 1983). It was dispersed towards west-northwest (Fig. 5a), and has a maximum thickness of ~ 1.5 m about 1.0 km from the La Fossa summit (s 134 in Fig. 1). In S33 (Fig. 2), the upper part of PAL B is an alternation of 3–4 cm of grey to brown ash layers. The sequence of ash and lapilli layers in PAL A and PAL B does not reveal the presence of either intra-sequence erosive unconformities or the interposition of reworked material. In contrast, subtle secondary volcaniclastic deposits occur between PAL B and PAL C1. PAL C1 consists of a plane-parallel sequence of ash and fine lapilli with no interposition of intra-sequence reworked material, suggesting it occurred without significant intra-eruptive pauses. At the trench site, PAL C1 is ~ 1.5 m-thick and consists of plane-parallel, mm to cm-spaced layers of fine to coarse dark ash bearing micro-vesicular, glassy juvenile pyroclasts. Moving upwards in the sequence, PAL C2 and PAL C3 show an increasing number of erosive unconformities. This indicates that Vulcanian explosions became more intermittent and discontinuous during this phase. PAL C2 is a 65 cm-thick, stratified, primary black to pinkish ash layers interbedded with cm-thick reworked sand beds. Within PAL C3, 2 main layers of reworked material (30–40 cm in thickness each) at the base and at the top are present, intercalated by a grey, primary ash layer.

At the trench location, PAL D is a 20 cm-thick, clast supported lapilli bed consisting of honey-colored, moderately vesicular lapilli, topped by sparse dm-sized, dark-colored bombs. PAL D represents the second marker bed (Figs. 4, 5b) in the southern sector of La Fossa cone (Frazzetta et al. 1983). PAL D reaches a thickness of 2 m at higher elevations on the cone southern flanks and exhibits an overall normal grading with the exception of scattered outsized bombs on its top. Overall, the PEU sequence is commonly exposed along gullies on the northern and southern sectors of the La Fossa cone, where it shows evidence of erosion and cross-bedding.

The Palizzi succession also includes 4 lava flows (see Fig. 1); from the oldest to the youngest, these are the following: Punte Nere lava delta (AD 1000–1170; Malaguti et al. 2022; trachytic in composition; Keller 1970, 1980), the highly viscous Commenda coulée (AD 1150–1350; Arrighi et al. 2006; rhyolitic in composition; Keller 1970, 1980), the Campo Sportivo lava tongue (undated; trachytic in composition; Keller 1970, 1980) and the Palizzi lava tongue (AD 1240–1300; Malaguti et al. 2022; trachytic in composition; Keller 1970, 1980). Based on the stratigraphic and chronological reconstruction of Malaguti et al. (2022), the Punte Nere lava delta was erupted either contemporaneously to PAL C or between PAL C and PAL D, while the eruptions of the Commenda lava and the Campo Sportivo and Palizzi lavas post-dated PAL D and are associated with stratified light-grey and dark-grey ashes interbedded between PAL D and the Breccia di Commenda (PAL E, PAL F, PAL G in Fig. 3; see also Fig. 2a in Malaguti et al. 2022).

### The Commenda (CEU) sequence

**Fig. 6:**
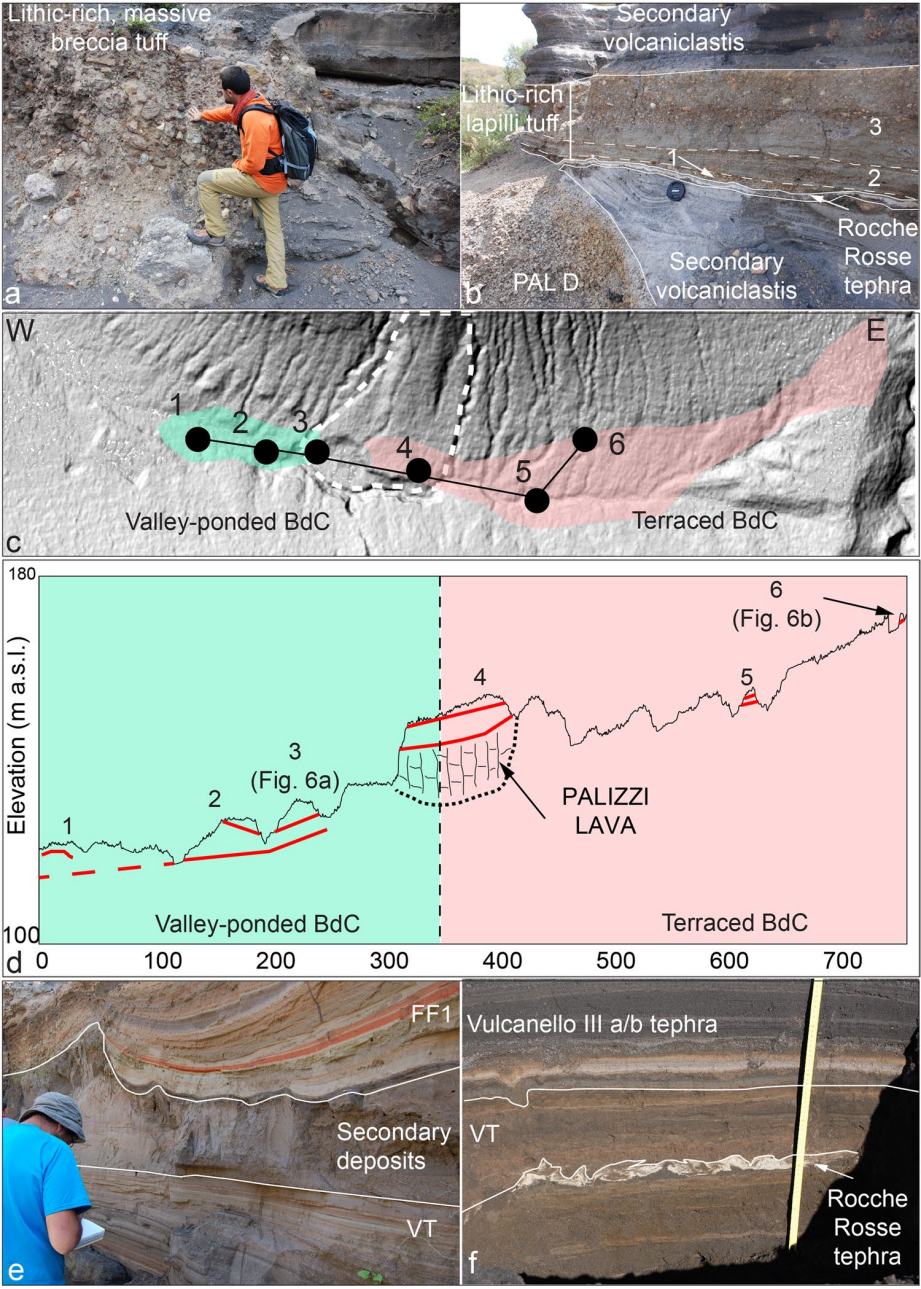
**a** Valley-pond, massive facies of the dense-PDC deposit. **b** BdC sequence in a fluvial terrace of the Palizzi valley (S33). **c** Map delineating the distribution of the diverse facies (terrace-based BdC and Valley-pondend BdC) showing the path of profile of panel d. **d** schematic representation of the relationship between the BdC facies (within red lines) and the Palizzi lava (in violet) in the Palizzi valley. The varicolored tuff sequence as observed in **e** medial location (Palizzi valley), separated from FF 1 by reworked deposits (S84), **f** distal location (Vulcanello platform)

After the deposition of the PEU sequence, the eruptive activity of La Fossa resumed with its most violent explosive and long-lasting event of the last 1000 years, which emplaced the Commenda Eruptive Unit (CEU) above a well-developed unconformity in the stratigraphic sequence (EU3; corresponding to S2 in Di Traglia et al. 2013). Only a brief description of the lowermost deposits (Breccia di Commenda—BdC) is reported here as it has been the focus of two recent works (Gurioli et al. 2012; Rosi et al. 2018). A major characteristic of the event is that it started contemporaneously with the Rocche Rosse eruption, which occurred ~ 10 km north on the nearby island of Lipari (Pistolesi et al. 2021). The BdC sub-unit can be divided into several layers (Fig. 6), from base upwards: (i) two, < 1 cm-thick, grey, fine ash layers, rich in millimetric ash pellets (Fig. 6a); (ii) a widely dispersed, coarse, fines-poor, crumbly (i.e., constituted by the presence of dense clasts, with grain-size ranging from lapilli to breccia, and the almost total absence of ash matrix), lithic-rich breccia (Fig. 6a, b); (iii) a moderately dispersed, dune-bedded, poorly sorted, coarse ash to lapilli (Fig. 6a, b); and (iv) a valley-confined, unconsolidated, matrix-rich, deposit, rich in decimetric to metric, oxidized lava and tuff blocks (Fig. 6a, b). The BdC sub-unit is overlain by up to several meters of multicolored, fine-grained, thinly stratified ash deposits. We interpret the (ii) sub-unit as resulting from an extremely violent, widely dispersed, blast-like PDC which overrun to the south the caldera cliff and spread northwards up to Vulcanello: pockets of coarse deposits with a few cm-sized clasts were observed on Vulcanello, > 2 km north of the present La Fossa crater.

The analysis of the stratigraphic and geometric relationships between the Breccia di Commenda deposits and the Palizzi lava within the homonymous valley (Fig. 6) shows that, west of the intersection between the lava flow and the valley, the deposits are valley-ponded accumulations with thicknesses of up to 3–4 m. To the east of the intersection point between the flow and the valley, the deposit is instead visible with relatively uniform thicknesses 1–2 m and situated at higher elevations compared to the outcrops to the west of the lava flow. The Breccia di Commenda eruption was followed by long-lasting mild ash emission which emplaced multicolor (reddish, brownish, and greyish), fine-grained, lithic, ashfall layers, named varicolored tuffs (VT; Fig. 5e, f, g; Capaccioni and Coniglio 1995). The ash is largely composed of hydrothermally altered minerals and the corresponding deposit is massive to parallel-bedded, and exhibit accretionary lapilli, plastic deformation, small-scale erosion channels, and gravitational slumps. The accumulation of tens of meters of VT on the upper part of the La Fossa significantly contributed to the growth of the cone (Fig. 5e). In medial areas, around the cone, reworked deposits and an erosive unconformity, separate VT from a similar but younger ash unit (FF 1; Fig. 6e). The ash deposits were dispersed in all directions with a predominance toward the east: 1.5 km east from the present crater (Caruggi area), VT attains a thickness > 2 m, whereas to the north (Vulcanello platform about 2.5 km N) it is only a few dm thick (Fig. 6f).

### The Fossa–Forgia Vecchia (FF) sequence

**Fig. 7:**
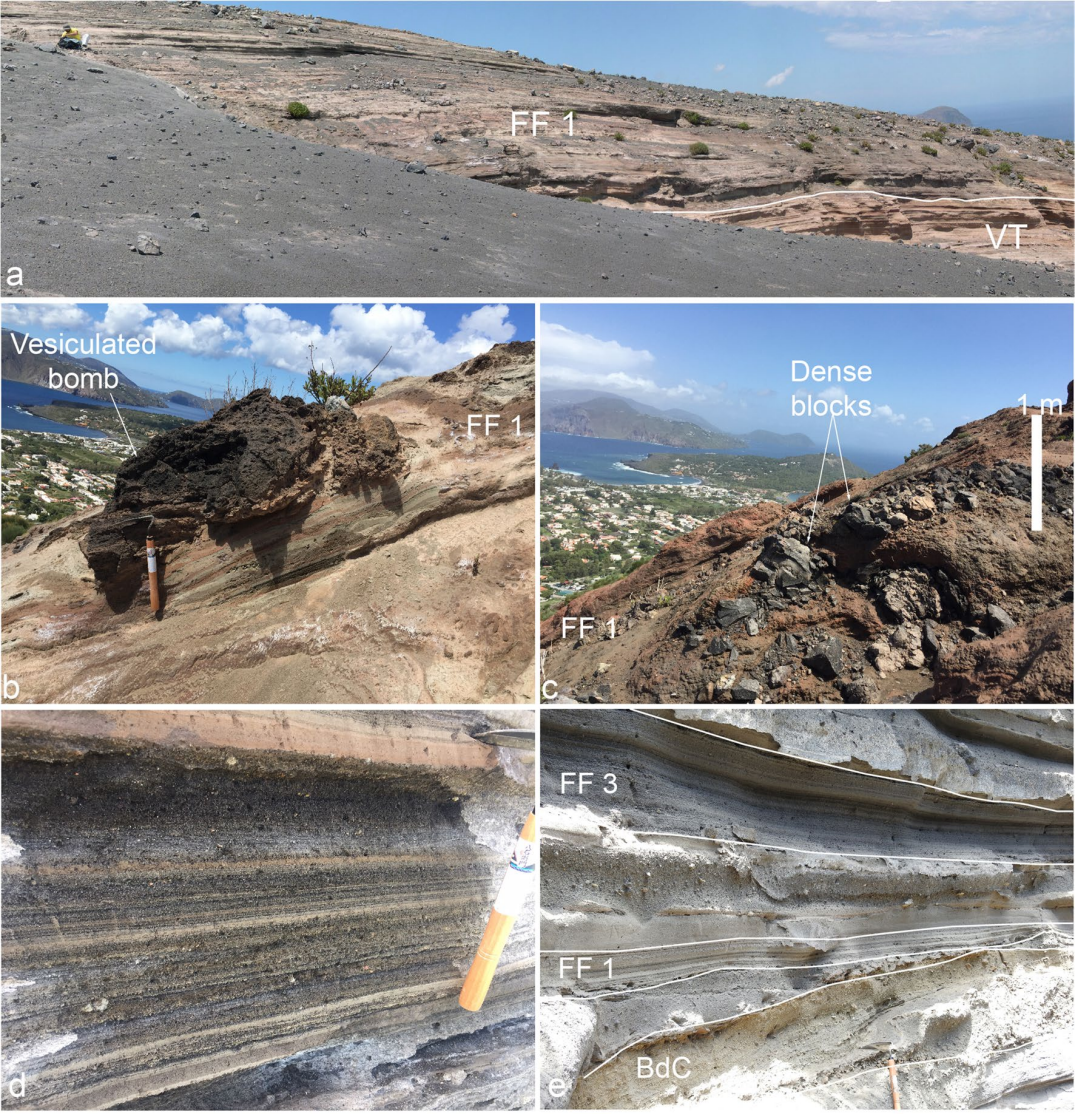
Main field characteristics of the Fossa–Forgia Vecchia deposits: **a** FF 1 (maximum observed thickness ~ 8 m) separated from VT by an unconformity (s69; the seated person acts as a scale); **b** vesiculated bomb and **c** ballistic block within the FF 1 sequence (northern flank of the La Fossa cone); **d** upper part of the FF1 sequence; **e** volcaniclastic sequence in the Palizzi valley exhibiting the alternation of primary volcaniclastic deposits of BdC, FF 1, and FF 3, interspersed with secondary volcaniclastic deposits

The base of the FF sequence is marked by an erosional unconformity (Fig. 7a; EU4, corresponding to S3 in Di Traglia et al. 2013) recognizable in most areas of the caldera. On the La Fossa cone flanks, the unconformity coincides with gullies or rills cut in the VT ashes. In some areas, the unconformity is overlain by a pinkish to brown, oxidized crust few mm-thick, likely arising from the hardening of tephra produced by acid dews from the fumarolic activity (see Fulignati et al. 2002). On the northern sector of the Vulcanello peninsula, EU4 unconformity is associated with a dark brown, dm-thick palaeosol, underlying Vulcanello III deposits and dated by Malaguti et al. (2022) at AD 1420–1460. The lowermost deposits of the Fossa–Forgia Vecchia sequence is preserved as multicolored, fine ash deposits with embedded blocks and bombs alternating with coarse bomb-rich deposits at the top of the La Fossa cone. This grades up into several metres of ash and lapilli deposits on the cone surroundings (Fig. 7). Deposits have been split into 5 eruptive episodes labeled as FF 1 to FF 5 (Figs. 2, 3, and 7).

The oldest unit (FF 1) namely consists of fine-grained ash, even close to the vent (Fig. 7a), and the color of the ash ranges from light-grey to red, to dark brown. On the La Fossa cone, the FF 1 sequence embeds scattered block and bombs up to one meter in diameter, often associated with impact structures (Fig. 7b, c). Moving upwards in the sequence, the multicolored fine ash becomes interbedded with dark ash and lapilli beds (Fig. 7d). This same vertical variation is visible in all the sections of the Palizzi valley at the foot and on the east flank of the cone. On the cone flanks and surroundings, FF 3 and FF 5 consist of parallel, thinly bedded, sequences of dark-colored lapilli and ash (Fig. 7). Each ash bed is massive to normally graded and is composed of dense glassy to moderately vesicular, grey lapilli, and subordinate reddish lava lithics. Where steep radial creeks of the western sectors of the cone join the main Palizzi valley, the primary FF 3 and FF 5 tephra packages are interbedded with massive to wavy-bedded, sand and pebble deposits (Fig. 7e). Higher up on the Fossa cone, the proximal deposits of the FF 3 and FF 5 consist of up to several meters-thick, thickly stratified, lapilli beds and coarse breccia deposits made up of dark angular lava blocks and black vesicular bombs. The striking feature of this eruptive phase is the activation of two eccentric vents on the northern flank of the cone (Forgia Vecchia area; FF 2 and FF 4 units). Materials ejected by these two vents consist of highly weathered lithic material, whitish in color for the older (FF 2) and reddish for the younger (FF 4; Fig. 3). A few outcrops recognized at Porto di Levante and Vulcanello suggest the dispersal of the FF 2 deposit might be considerable and possibly resulting from a lateral phreatic blast.

### Pietre Cotte (PC) sequence

**Fig. 8:**
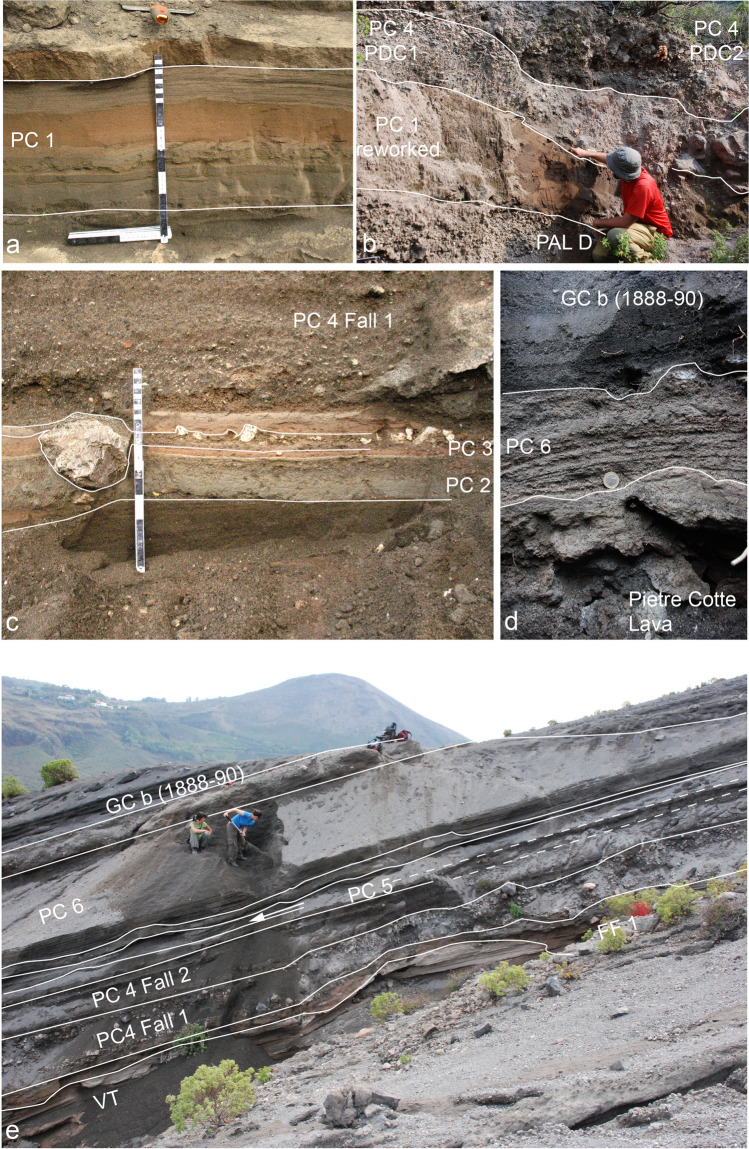
Main sections used for the study and labelling of the deposits of the PC sequence. **a** and **b** In the Palizzi Valley, located west of the Palizzi lava flow, it is possible to observe the occurrence of a stratified tephra layer consisting of fine to coarse ash (PC 1) within a sequence of secondary volcaniclastic deposits; **c** and **e** natural cuts on the eastern flank of the La Fossa cone; **d** stratigraphic relationship between PC 6 and the underlying block bed topping the Pietre Cotte lava

**Fig. 9:**
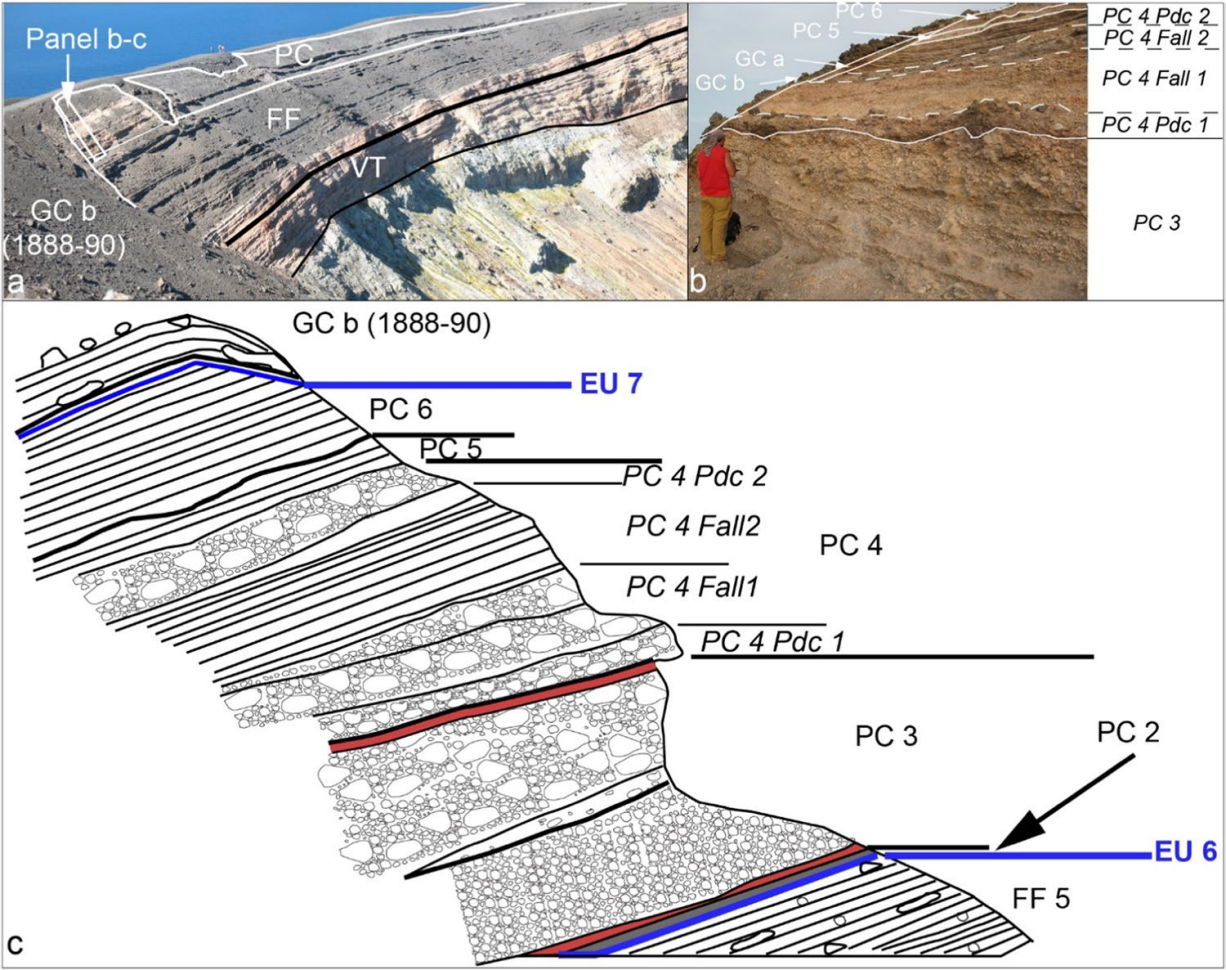
**a** Proximal sequence of La Fossa cone products, the sequences CEU, FF, and PC. The Gran Cratere (GC) sequence, relating to the final part of the 1888–1890 activity, crops out in clear unconformity with respect to the other sequences suggesting the change of vent location during the volcanic activity; **b** details of the crater rim tephra sequence; **c** simplified diagram of the upper part of the crater rim of the Fossa cone

The FF and Pietre Cotte (PC) deposits are separated either by an erosional unconformity (EU5) or by a thick accumulation of secondary volcaniclastic deposits 3–7 m thick. These deposits primarily comprise fragments originating from tephra packets FF 1 – FF 3 – FF 5. Both features indicate a significant period of volcanic dormancy lasting several decades. Within these deposits, a distinct layer (PC 1) is identified at a site located 1 km south of the 1888–1890 crater (refer to Fig. 8a, b) consisting of approximately 30 cm of reddish to grey fine ash. The maximum thickness is observed south of the crater suggesting that it was dispersed south by prevailing winds. PC tephra sequence is composed of packages associated with at least six major eruption pulses (Figs. 2, 3, 8, and 9). PC 1 is bounded at its base and top by two notable unconformities (EU5 and EU6), indicating that it represents an isolated event between FF 5 and PC 2. Nevertheless, we have included it within the PC sequence.

The thickest and most complete section of the sequence crops out along the steep walls of the 1888–1890 crater. The truncation of the outwards dipping tephra sequence resulted from the widening of the 1890 crater due to collapse events during the eruption (Fig. 9). Further stratigraphic sections were examined on the eastern flank of the Fossa cone and in the cone’s surroundings. As a whole, they added further important information on presence of erosional unconformities or reworked deposits and on the dispersal of each bed (e.g., s65 and s92 in Fig. 2).

**Fig. 10:**
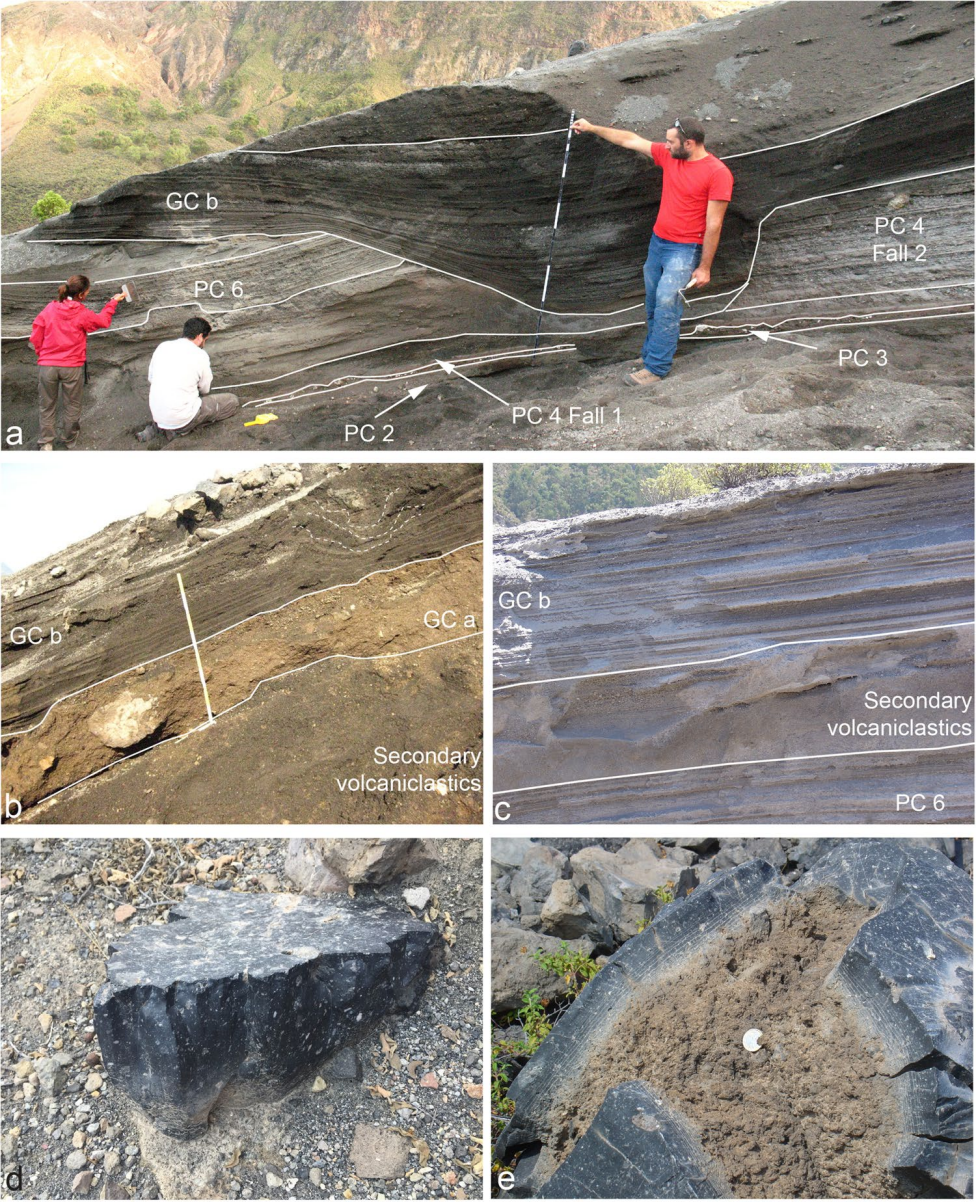
**a** natural cuts on the eastern flank of the La Fossa cone, where it is possible to study the stratigraphic relationship between the GCb deposits and the PC sequence; **b** orange-colored unit GCa, made up of lithic material, topped by the 1888–1890 dark-grey, ash and lapilli; **c** GCb and PC 6, with interposed secondary volcaniclastic deposits, on the SE flank of the La Fossa cone, which show internal parallel-bedding; **d** dense jointed juvenile block, and **e** a bread-crusted bomb expelled during the final phase of the 1888–1890 eruption, exhibiting both a vesicular interior and a dense glassy rind. Within the glassy rind, fine lineations likely result from incremental expansion of the bomb during its fly

From base to top, PC 2 is a massive, cohesive, greenish, fine ash layer with scattered white pumiceous lapilli and greyish, black and red lava lithic clasts (Fig. 8c). It reaches 20 cm thick on the south-west flank of the cone, but the deposit is rather ubiquitous around the cone with limited thickness variations. The crater wall section (Fig. 9) includes two thicker tephra packages (PC 3 and PC 4; Fig. 9b). PC 3 consists of a pink, fine ash overlain by three coarse-grained beds: (i) a lower meter-thick, clast-supported pumice deposit, made up of pumice lapilli and bombs and fresh to oxidized angular lava clasts (Fig. 9b) overlain by (ii) a bomb-supported, reversely graded, deposit constituted by white pumice clasts, banded pumiceous-obsidian clasts and breadcrust bombs with thin crusts, that we interpret as a fallout from sustained explosive activity, and by (iii) a pink, cm-thick ash bed which seals the sequence at its top (Fig. 8c). Correlation of the PC 3 with sections at the foot of the cone indicates that the overall dispersal direction is southwards. At the crater-rim section and along the southern flank of the cone, west of the Palazzi lava flow, a sheet of poorly sorted deposits forms lobes with thicknesses varying between 1 and 1.5 m (PC 4 PDC1; Fig. 8b). These deposits consist of matrix-supported breccias, rich in highly altered lava blocks, with a lithology similar to the Commenda lava. PC 4 Fall 1 is a massive, clast-supported deposit composed of lapilli and bombs, with a scarce ash matrix, rich in white to light grey pumice lapilli and bombs, with subordinate obsidian clasts that we interpret as a fallout from sustained explosive activity (Figs. 8, 9). It is overlain by parallel-bedded alternations of coarse ash and lapilli beds containing black obsidians and scoriae, with rarer grey pumices (PC 4 Fall 2; Figs. 8, 9). The dispersal axis of PC 4 Fall 1 is probably south-eastwards (Fig. 4c). On the southern, mid-lower sector of the cone, both west and east of the Palazzi lava flow, there is a laterally continuous, massive, poorly sorted deposit forming lobes with thickness varying between 1 and 1.5 m just below the present ground level (PC 4 PDC2; Fig. 8b). Locally, the lower part the unit consists of a matrix-supported breccia rich in lava blocks whereas the upper part is clast- to matrix-supported, and almost entirely made of pumice clasts with occasional bread-crust bombs and block. Obsidian blocks are porphyritic with alkali feldspar phenocrysts and centimetric grey-colored, porphyritic enclaves similar to those present in the Pietre Cotte lava flow (Piochi et al. 2009). In the crater wall section, PC 5 consists of an 80 cm thick, clast-supported deposit made up of dm-sized obsidian lava blocks and pumice bombs which overlies an erosional surface, that we interpret as a fallout from explosive activity. On the eastern flank of the cone, the bed is around 10 cm thick, and consists of a package of parallel-bedded, ash and lapilli sandwiched between reworked deposits that overlie PC 4. The topmost deposit (PC 6; Fig. 10a, c) consists of thinly bedded, plane parallel layers of ash and fine lapilli. The greatest thickness is present on the eastern and south-eastern slopes of the cone (2.5 m) where the deposits are sandwiched between debris flow deposits or an erosive surface cut into the underlying Pietre Cotte succession. A stratigraphic trench dug in the lowermost, flat top, of the Pietre Cotte lava flow, revealed the presence of about 15 cm of ash and lapilli attributable to PC 6 directly emplaced on the lava blocks and sealed at top by the 1888–1890 tephra (Fig. 8d). PC 6 is mostly dispersed towards the east of La Fossa cone (Fig. 5d).

### The Gran Cratere (GC) sequence

The time break between the PC sequence and the deposits associated with the nineteenth century eruptions (GC) is marked by a further erosional unconformity (EU7). The GC sequence includes the deposits of the last two eruptions of the La Fossa cone (Fig. 10), with the youngest that can be unequivocally attributed to the 1888–1890 eruption (Mercalli and Silvestri 1891). The deposits of the first eruption (GCa) crop out mostly on the northern, upper slope of the La Fossa cone, in cuts crossed by the path that climbs to the crater, and on top of the upper part of the Pietre Cotte lava flow. Thinner deposits at the same stratigraphic level are encountered on the eastern and southern slopes of La Fossa. These deposits consist of clast- to matrix-supported breccia beds rich in oxidized blocks (15–20 cm diameter) of flow-banded, fresh to strongly altered glassy (rhyolitic) lavas (Fig. 10b). The deposits are laterally variable in thickness and lithology, reach several tens of cm in thickness on the cone and rapidly thin out outward and disappear at the base of the cone. The orange-color of the breccia clasts suggests oxidation of the outer surface resulting from the reaction of hot hydrothermal, material with the oxygen of the atmosphere.

**Fig. 11:**
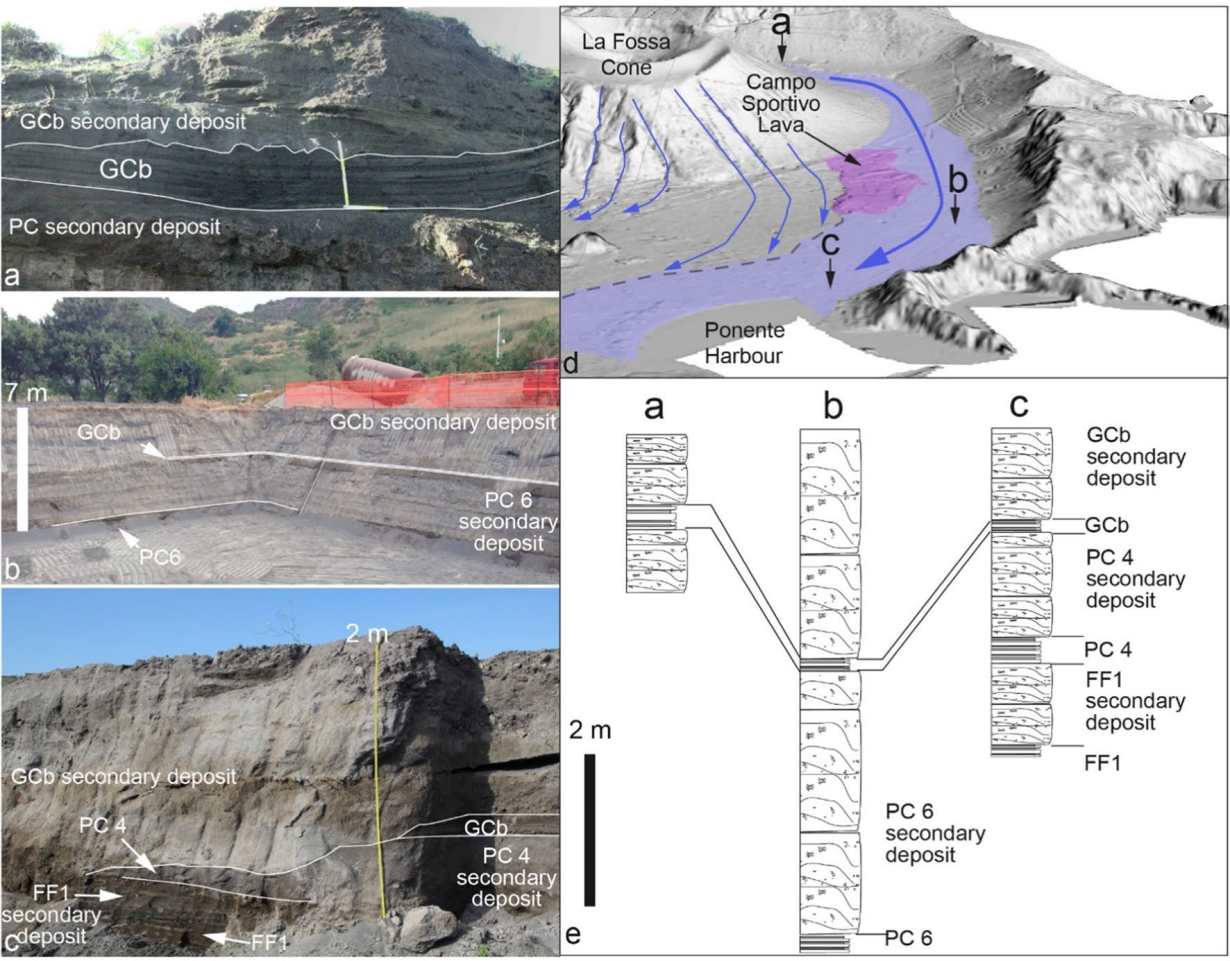
The sequence of primary (FF1, PC 4, PC6, GCb) and secondary volcaniclastic deposits is observable across the following locations on Vulcano Island: **a** natural outcrop in the Palizzi valley, located south of the La Fossa cone; **b** excavation site in the eastern part of the Vulcano Porto plain, which was excavated for the island’s desalination plant; **c** excavation near Ponente Harbour, one of the two designated ports for the island's evacuation in case of emergency. In map **d**, the positioning of panels **a**, **b**, and **c** relative to the Palizzi valley and the Campo Sportivo lava flow can be observed. The lava flow in question acted as a natural barrier, resulting in the constriction of sediment-laden water flow (i.e., debris floods) to a narrow area, thereby channeling the sediments towards Ponente Harbour. The diagram features primary flow lines, distinguished in blue, derived from Bonasia et al. (2022). In **e**, stratigraphic logs corresponding to panels **a**, **b**, and **c** are shown

Moving upwards, the GCb sequence consists of a parallel-bedded alternation of fine to coarse ash, lapilli and bombs that crops out at many natural cuts around the cone and constitutes the youngest tephra of La Fossa activity associated with the 1888–1890 eruption. The most proximal and complete, 230 cm thick, tephra sequence was studied in a hand-dug trench about 500 m east of the present crater. The lower 130 cm consist of alternating parallel-bedded, fine to coarse ash layers up to 7–8 cm thick, while the upper 1 m consists of parallel-bedded, ash and lapilli layers which become richer upwards in variably vesicular bombs and then in blocks up to 10 cm in diameter. Larger bombs and blocks are often underlain by impact sags. The very upper part of the 1888–1890 deposit consists of a carpet of dense blocks and subordinate breadcrust bombs (Fig. 10e) that mantel the upper part of the La Fossa cone. Dense, glassy blocks, often show evidence of cooling joints orthogonal to the outer surface (Fig. 10d). The breadcrust bombs have glassy rinds ranging in thickness from less than 1 cm to a few cm. Some blocks display an internal brecciated texture. Moving away from the crater area, the 1888–1890 tephra sequence rapidly thins and the overall grain-size of the deposits decreases, while remaining parallel-bedded (Fig. 10c; Fig. 11a); some key layers, or alternations of packages of layers can be traced between different outcrops. On the southern sector of La Fossa, outside the caldera border, the total thickness of the sequence is 20–30 cm.

In the Vulcano Porto area, along the extension of the Palizzi Valley towards Ponente Harbor (Fig. 11b, c), the thickness of GCb is approximately 10 cm. This tephra layer underlies and is partly interbedded with secondary volcaniclastic sequences (interpreted as intra-eruption lahar deposits). The thickness of the post-GCb sequence exceeds 2 m, whereas the thickness of the stratified, secondary deposits resulting from the reworking of PC6 tephra attain about 2.4 m (Fig. 11b). In an excavation conducted near s154 (Fig. 11c), the thickness of the same secondary volcaniclastic deposits post-GCb measures approximately 2.4 m.

### The Vulcanello sequence

**Fig. 12:**
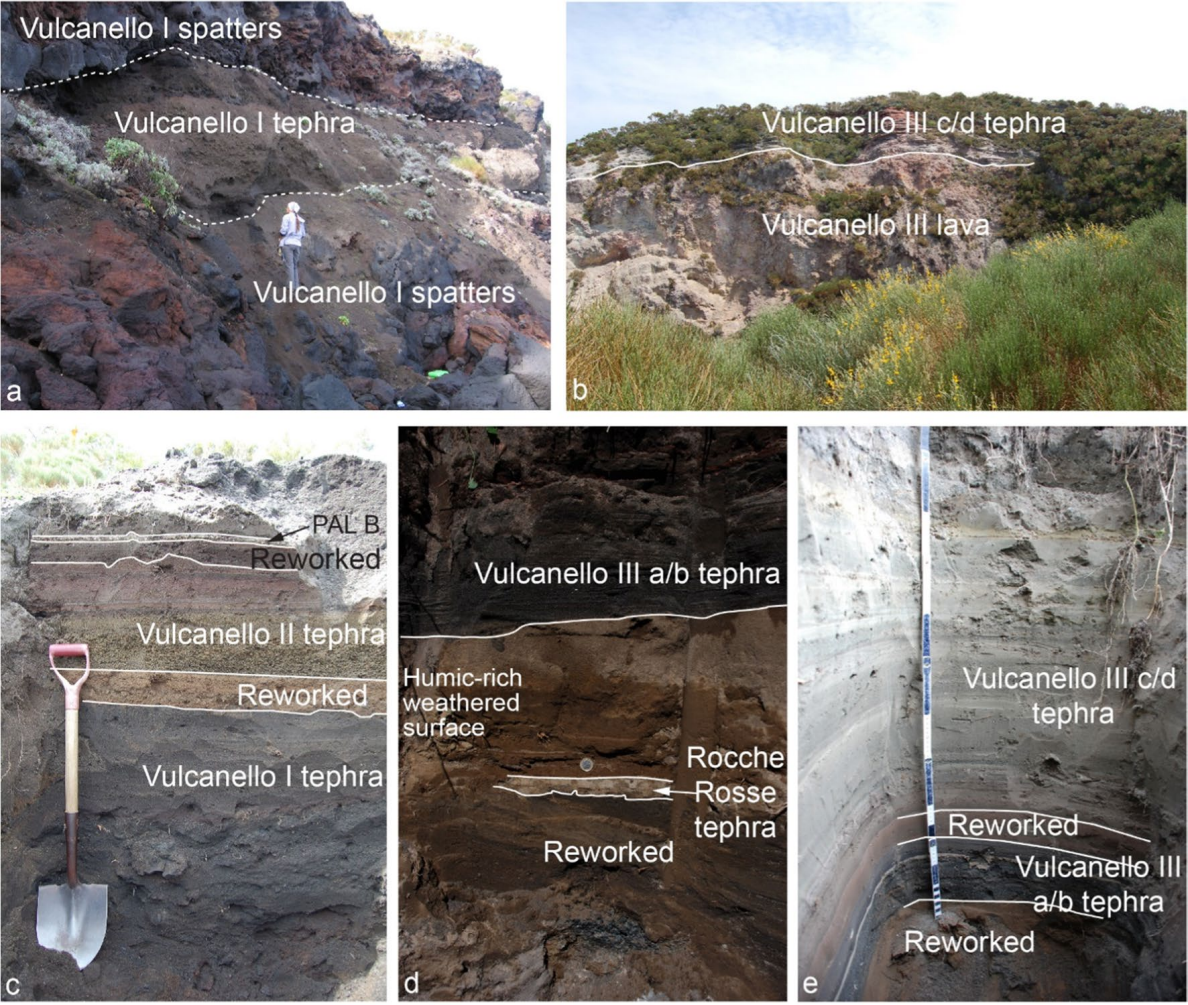
**a** Vulcanello cliff, where the presence of multiple spatter beds and loose tephra beds associated with the construction of the lava platform and the first cone is shown; **b** internal view of the third crater of Vulcanello, where it is possible to observe the unconformity between the final tephra (Vulcanello III c and d in Fusillo et al. 2015) and the underlying lavas (Punta del Roveto and Valle dei Mostri lava flows); **c** Sect. 42, located at E close to the coastal cliff, shows a sequence of tephra from the first and second cones of Vulcanello, separated by reworked deposits, and covered by the PAL B level from the Fossa cone; **d** Sect. 86 (coin for scale) shows a sequence of tephra constituted by (from bottom to top) a secondary volcanoclastic sand, a fine rhyolitic ash layer (6 cm) related to the Rocche Rosse tephra (AD 1240–1300; Malaguti et al. 2022) from Lipari, a paleosoil rich in organic fragments, dated to AD 1420–1460 (Malaguti et al. 2022), covered by a stratified ash sequence related to the Vulcanello 3 (a/b) activity (likely related to the Punta del Roveto and Valle dei Mostri lava flows; Malaguti et al. 2022); **e** Sect. 85 showing the tephra associated with the third cone (Vulcanello 3), separated from each other by reworked deposits

Volcanic activity at Vulcanello produced a series of lavas and three overlapping cones along with minor tephra units (Davì et al. 2009; Fig. 12). Fusillo et al. (2015) interpreted the sequence of tephra and lavas associated with this activity as mainly subaerial and minor submarine and proposed that it occurred in two clusters of eruptions. Here, we describe the three main stratigraphic sections that are useful, *inter alia*, for the correlation between the activity of Vulcanello and that of La Fossa cone, thus allowing to strengthen the chronological reconstruction and the relative timing between the two eruptive centres of La Fossa caldera.

The first cluster (AD 900–1040; Malaguti et al. 2022) had two distinct phases (Vulcanello 1 and 2; Fusillo et al. 2015) and was punctuated by a break marked by reworked deposits (Fig. 12c). The construction of Vulcanello 1 cinder and spatter cone was associated with the formation of the main subaerial lava platform. The construction of the Vulcanello 2 cinder cone was linked to the emplacement of a submarine pillow lava field (VO6 in Romagnoli et al. 2013; Fusillo et al. 2015). Tephra and lavas of the first cluster are ubiquitously overlain by 1–2 cm-thick, rhyolitic pumice lapilli bed (PAL B), thus indicating that PAL A and Vulcanello 1 and 2 were pene-contemporaneous.

The tephra beds related to the second cluster (Vulcanello 3) were identified in several trenches dug on the lava platform. They rest on a weathered surface formed on top of La Fossa VT ash which contains scattered charcoal fragments (Fig. 10d) dated with ^14^C to AD 1420–1460 (Malaguti et al. 2022). This activity (Vulcanello 3) had two distinct phases, as indicated by a thin humic-rich, weathered surface in between. The first phase includes dark-colored, ash and lapilli layers produced by Vulcanian activity (Vulcanello IIIa and IIIb in Fusillo et al. 2015) and two lavas: Roveto lava (AD 1400–1450) and Valle dei Mostri lava (AD 1400–1470; Malaguti et al. 2022). The second phase (Vulcanello IIIc and d in Fusillo et al. 2015), the deposits of which were emplaced above the youngest humic-rich, weathered surface with a ^14^C age of AD 1450–1650; (Keller 1970), built a small tuff cone without significant shift in vent location. This was characterized by alternating Vulcanian and phreatic activity, which deposited coarse to very fine, grey, red, and whitish ash layers, containing scattered brown to black spatter clasts (Fig. 12b, e).

### Correlation of tephra sequences with historical chronicles

The correlation between the observed tephra packages and historical eruptions described in the chronicles was based on the following evidence: (i) duration of the eruption (single vs multi packages of tephra); (ii) intensity of the eruption/individual explosions; (iii) deposit dispersal; (iv) vent location; (v) physical features of the erupted material. Below we discuss these from the youngest to the oldest (Table 1).

**Table 1:** Schematic diagram illustrating the assignment of ages and the interpretation of the aggressive eruptive style and primary phenomena, reconstructed through the intersection of field-based data and historical chronicles

Unit	Age	Interpretation
GCb	1888–1890	Vulcanian eruption from La Fossa cone. Tephra fallout and ballistic projectiles
GCa	1873 (and/or 1888)	Phreatic explosions from La Fossa cone. Tephra fallout, PDCs, ballistic projectiles
PC 6	1786	Vulcanian eruption from La Fossa cone. Tephra fallout and ballistic projectiles
PC 5	1775	Vulcanian eruption from La Fossa cone. Tephra fallout and ballistic projectiles
Pietre Cotte lava	1771 or 1775	Rhyolitic coulee
PC 4	1771	Vulcanian eruption from La Fossa cone, with phreatic onset. Tephra fallout, PDCs, ballistic projectiles
PC 3	1739	Vulcanian eruption from La Fossa cone. Tephra fallout
PC 2	1731	Vulcanian eruption from La Fossa cone. Tephra fallout
PC 1	1631	Phreatic explosions from La Fossa cone. Tephra fallout
Vulcanello III c/d	AD 1450–1650	Vulcanian and phreatic eruption from Vulcanello. Tephra fallout and ballistic projectiles
FF a 5	1550	Vulcanian eruption from La Fossa cone. Tephra fallout and ballistic projectiles
FF 4		Phreatic explosions from Forgia Vecchia eccentric vents (La Fossa cone). Tephra fallout and ballistic projectiles
FF 3		Vulcanian eruption from La Fossa cone. Tephra fallout and ballistic projectiles
FF 2	1444	Phreatic explosions from Forgia Vecchia eccentric vents (La Fossa cone). PDCs, ballistic projectiles
Vulcanello III a/b	AD 1400–1470	Vulcanian eruption from Vulcanello. Tephra fallout, ballistic projectiles, lava flows
FF 1	1403 (?)	Vulcanian and phreatic eruption from La Fossa cone. Tephra fallout and ballistic projectiles
Varicolored tuffs		Phreatic eruptions from La Fossa cone. Tephra fallout
Breccia di Commenda	AD 1240–1300	Phreatic eruption from La Fossa cone. Blast-like diluted PDCs, column-collapse dense PDCs, ballistic projectiles, tephra fallout
Vallone Palizzi lava/PAL G	AD 1240–1300	Vulcanian eruption from La Fossa cone. Tephra fallout, lava flows
Campo Sportivo lava/PAL F		Vulcanian eruption from La Fossa cone. Tephra fallout, lava flows
Commenda lava/PAL E	AD 1150–1350	Vulcanian eruption from La Fossa cone. Tephra fallout, lava flows
PAL D		Sub-Plinian eruption. Tephra fallout
Punte Nere lava/PAL C3	AD 1000–1170	Vulcanian eruption from La Fossa cone. Tephra fallout, lava flows
PAL C2		Vulcanian eruption from La Fossa cone. Tephra fallout
PAL C1		Vulcanian eruption from La Fossa cone. Tephra fallout
PAL B		Sub-Plinian eruption. Tephra fallout
Vulcanello I/II	AD 900–1040	Strombolian/Hawaiian eruptions. Lava flows, tephra fallout, ballistic projectiles
PAL A	AD 900–1020	Vulcanian eruption from La Fossa cone. Tephra fallout

Deposits of the 1888–1890 eruption can be easily traced around the cone as they are the most recent tephra underlying the present ground level or post-eruptive debris flow deposits. The 1888–1890 eruption’s products consist of 2–3 m of ash and lapilli beds (which constitute the eruption’s main volume) as shown by the trench that we dug about 500 m east of the crater and by the overlaying the ballistic blocks and bombs field on the cone’s upper portion.

GCa represents a sequence of lithic-rich layers attributed to the phreatic activity preceding the 1888–1890 eruption. The two most significant eruptions took place from 7 September 1873 to 27 October 1873, and on 3–5 August 1888. The first event, documented by Mercalli and Silvestri (1891) as well as by De Fiore (1922), described upon the accounts of Picone, was reported to have originated from four distinct vents during the eruption on the northern rim of La Fossa crater. The description of the activity agrees well with the presence of coarse breccia and PDCs deposits on the northern slope of the volcanic cone. Salino (1874) considers this event responsible for the sedimentation of a layer of ash over the entire area surrounding the cone, which was subsequently heavily eroded by post-eruptive rainfall. In contrast, the phreatic phase preceding the 1888–1890 eruption ejected abundant ballistic blocks and a thin layer of ash (Mercalli and Silvestri 1891). Based on our current understanding, it is not possible to clearly distinguish the deposits of the two phreatic phases in the field as they occupy the same stratigraphic position, and because they are composed of similar altered material, as described by Mercalli and Silvestri (1891). Therefore, we consider the GCa level as a cumulative record of what can be regarded as a series of phreatic explosions leading up to the 1888–1890 eruption. However, based on descriptions, duration of the activity and field observations, most of the deposits, and particularly those emplaced on the northern flank of the volcanic cone (breccias + PDCs), can be confidently attributed to the 1873 eruption.

PC 6 tephra features and areal distributions (Fig. 4) are in excellent agreement with the description of the 1786 explosive activity reported by Spallanzani (1792–1795) (“*Eruption on 12 January 1786 lasted for a whole month sending large quantities of ash and burning stones into the air*”). Spallanzani adds that “*the outcome of the arena was so great that the surrounding places remained highly covered by it, and to the east of the crater and in a short distance from it there is presently a conical mound with a circumference of half a mile, resulting from this pulverized substance, produced entirely, as they told me, under such circumstances.*” Several scientists visiting Vulcano at the beginning of nineteenth century confirmed the presence of an elliptical, E-W elongated, deep crater, in the central-northern sector of the Fossa cone left by the 1786 event.

The chronological attribution of the Pietre Cotte lava flow eruption has been a matter of debate in the literature. Some authors preferred the age of 1739 (De Fiore 1922; Arrighi et al. 2006; Piochi et al. 2009) while others that of 1771 (Mercalli 1883). Evidence that concurs to establish the age of this eruption can be summarized as follows: (i) the scientist De Luc (1780), who visited Vulcano in 1757, reports that he entered the crater through a deep creek opened on the northern flank of the cone. The creek later disappeared, and the only plausible explanation is that it was buried and obliterated by the emission of the Pietre Cotte lava, which should then post-date 1757; (ii) the Dutch painter Houel unequivocally drew the Pietre Cotte lava in one of his paintings in 1776–1778; (iii) Déodat Guy Silvain Tancrède Gratet de Dolomieu, the French geologist who visited the Aeolian Islands from 12 to 21 July 1781 and two years (1783) later, published an account of the journey under the title of “Voyage aux Iles de Lipari fait en 1781” (de Dolomieu 1783). Based on the information he collected, he attributed the outpouring of the Pietre Cotte flow to an eruption occurred in 1775. The description of de Dolomieu reports that “[…] *The last eruption, finally, of which I was able to gather news is that of 1775. It was accompanied by earthquakes which were strongly felt in the nearby islands. For several months a considerable crash was heard, underground thunder and all the other phenomena that characterize a great upheaval. The crater threw very large stones and blocks of glassy lava far away, many of which are still visible in the circular valley that surrounds the volcano; it spewed large quantities of white ash which covered the island of Lipari and was carried as far as Sicily*.” Therefore, during his brief visit to Vulcano and Lipari (one day for each island), de Dolomieu concluded that before the emission of the lava there was low-intensity explosive activity, even if he is not specific about who provided him with the information. From the perspective of the field observations, we do recognize a thin primary tephra that we have labeled as PC5, stratigraphically overlying some PC4 secondary deposits. The PC4 tephra sequence consist of a large number of beds and for this reason we believe it matches well with the the 1771 eruption that, as reported by Mercalli and Silvestri (1891) and by De Fiore (1922), was long-lasting, from 17 February to May (3 month), and had various phases, with different intensity and height of the eruption columns. The writings of Francesco Ferrara which in turn are based on information received from Abbot Trovatini residing in Lipari at the end of the eighteenth century, who refers that “*On 17 February 1771, the mountain unleashed a tremendously loud thunder, preceded by a powerful jolt that shook Lipari. The inhabitants, who were awakened (as it occurred at 2 o'clock in the night), witnessed the mountain hidden in black smoke and a column of fire rising from the crater. The southerly wind pushed all these materials, along with the smoke, in the form of a dark cloud that thickly flickered inside, covering the sky of Lipari and scattering a sustained and abundant rain of ash. The eruptions continued, always preceded by strong tremors, not only throughout that night but almost every day throughout the month of February. Layers of ash accumulated in Lipari, reaching several inches in height. The phenomena repeated almost every day in the following months of March and April, and nearly until mid-May. The smoke and earthy rain sometimes darkened the day in Lipari to such an extent that the islanders couldn't see each other at close distances. The accumulated ashes eventually reached such a thickness in Lipari that they covered the vegetation, causing significant harm to livestock. Occasionally, during that period, the eruption involved large masses of debris, which spread fear among the inhabitants of the island of Lipari. It was only in February that the eruptions were preceded by strong tremors, while in other times they were accompanied by rumblings and deafening noises that horrified the people of Lipari*.” The attribution of the Pietre Cotte lava to 1775 made by Dolomieu was somehow questioned by Mercalli who believed de Dolomieu had been misled by his (anonymous) source, which instead of 1775 should have reported 1771.

Based on the stratigraphic data and historical chronicles, we cannot get to a firm conclusion on the year of eruption of the Pietre Cotte lava flow. Both the final stages of the 1771 eruption or the year 1775 remain possible: indeed, the thin tephra layer of lapilli and ash (PC 5) on the southern flank of the cone that overlies the reworked deposit above the 1771 tephra seems to fit well with the 1775 event. Whatever the correct interpretation, the time frame of the lava eruption is in any case bracketed between 1771 and 1775.

Tephra unit PC 3 is attributed to the 1739 eruptions with the main phase occurred on March 9 and on May 4. De Fiore (1922), reporting what Mongitore refers to as observations made in the town of Naso (Sicily, 35 km SSW of Vulcano island), says that “*A white cloud expanded from Vulcano, which roared continuously like a battery of muskets exploding in the breach. And indeed, as such a cloud approached our districts it was seen throwing very numerous stones, one of which was still hot on the beach*.” It is interesting to note that the white color of the cloud, the sector of dispersal of the eruptive cloud, the frequent lighting activity and the convective carrying capacity of light pumice, match well with the upper, coarser bed. The explosion of March 9 could instead match with the lower, finer-grained pumice fallout bed. At the end of the tephra sequence, there is a lithic-rich PDC deposit, compatible with a phreatic explosion that occurred in this sequence.

The PC 2 deposit (fine-grained, greenish & pink-colored, cohesive ash), fits well with the 1731 eruption descriptions. The presence of altered ash is consistent with some considerations made by De Fiore (1922), which argues that “*all the aforementioned facts indicate a strong explosive eruption with a large projection of ashes, perhaps ancient materials: this seems to be deduced from the detail of the destruction of grass which is mostly and more easily operated by ashes coming from the crushing of ancient materials of the crater scaffolding, rich in acid products due to the fumarolic emanation; rather than from coeval autogenous ashes that have much less accentuated or completely zero caustic power*.”

It is not clear whether the few eruptions reported in seventeenth century (AD 1613) are attributed to La Fossa cone or to Vulcanello, which was active at that stage (Vulcanello 3) and emplaced deposits on a weathered surface dated at AD 1450–1650 (Malaguti et al. 2022). Mercalli and Silvestri (1891), as well as De Fiore (1922), suggest an eruption occurred on 24 August 1631 based on the historical accounts. Considering the stratigraphic position of PC 1, which is observed as a distinct layer between FF 5 and PC 2 and is comprised between two substantial sequences of reworked sediments (Fig. 7b), it is reasonable to attribute this deposit to the eruption of 1631. The fine ash layers of the PC 1 outcrop in the southern sector of the La Fossa cone and show a maximum thickness along the direction connecting La Fossa cone to the village of Naso in Sicily where ash fall is reported by historical reports.

Based on the stratigraphic evidence, La Fossa cone seems to have been quiescent during the latter half of the sixteenth century, in agreement with the marked erosion of the cone and the emplacement of secondary volcaniclastic deposits (Fig. 7b). Traces of eruptive activity are reported by Fazello, an Italian Dominican friar, historian and antiquarian. A possible eruption is described on Vulcano on the 5 February 1444, according to Fazello reporting in turn Pietro Ranzano (1427 or 1428–1492), another Dominican friar who was in Catania in 1444, who reports “*Mount Vulcano, which is one of the Aeolian Islands, contrary to the usual, on the 5th of the same month, February 1444, before dawn, hurled masses of flames and fiery rocks into the sky. Moreover, four of these innumerable boulders, very large, fell six thousand paces away from the same island with a terrible crash*” (translation from the Latin; see Ciuccarelli et al. 2019). According to this latter chronicle, the eruption of 5 February 1444 ejected ballistic blocks up to distances of ~ 11 km (a distance that was perhaps overestimated). The great distance reached by blocks could be suggestive of a lateral component in the ejection a fact that could match with the eruption of Forgia Vecchia 1 (FF 2), which is an eccentric, phreatic eruption, with the vent located on the north flank of the Fossa cone. The report of Alberti (1550) hypothesizes that the island of Vulcano in the sixteenth century had three distinct vents, compatible with the activity of Vulcanello, La Fossa and the eccentric craters of the Forgia Vecchia, for which a second phase of activity is recognized towards the end of the sequence of tephra Gran Cratere–Le Forge. We attribute to this sequence an age between the first half of the fifteenth century to the AD 1550 eruption, related to the FF 5 unit.

For the older sequences described here (Palizzi, Commenda, and Vulcanello 1 and 2), there is no description of specific events, even though in Al-Iddrisi's descriptions Vulcano was in strong activity (Yilmaz 2016) during the period in which he wrote Book of Roger (between AD 1095 and AD 1154). The Arab geographer reported: “*To the north of the Sicily Island, there is a number of islands that we need to describe one by one in this chapter. The first of these islands is an uninhabited island called Vulcano that burns constantly day and night*.” This is consistent with what was observed in the Palizzi and in Vulcanello 1 and 2 successions, associated to ash emissions (PAL A and PAL C, Vulcanello 1 and 2), more intense explosions with emplacement of pumice (PAL B and PAL D), and of lava flows, and also in agreement with age obtained by Malaguti et al. (2022) for the onset of the PEU sequence. A further confirm of an important eruptive activity between the end of the tenth century and the twelfth century, when Vulcanello and Vulcano were still two separate islands, is presented in Manni and Rosi (2021), who suggest that the isthmus connecting the two islands formed later, in the sixteenth century AD.

There are no descriptions of activity associated with the Breccia di Commenda and the varicolored tuffs, because the period was geopolitically unstable (De Fiore 1922). In particular, in 1544, the Sack of Lipari took place, when Khayr al-Dīn (known as Barbarossa, corsair and commander of the Ottoman fleet), sacked the island and enslaved all or almost all the inhabitants of the island. During this looting, the city's archives were destroyed, and many traces of past events were lost.

## Discussion

### Eruptive history of the La Fossa caldera in the last 1100 years

**Fig. 13:**
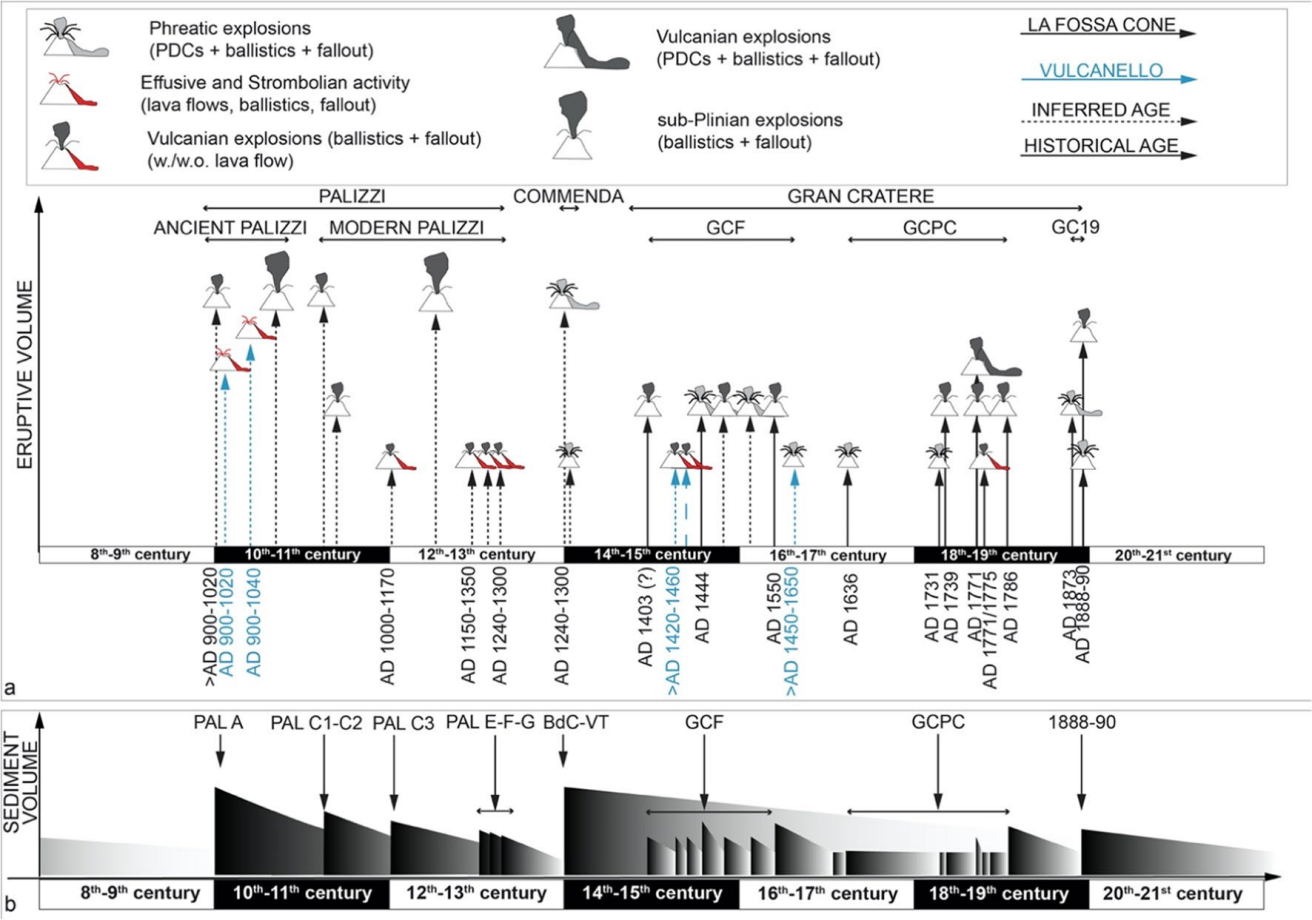
Summary diagram illustrating the overall eruptive history of La Fossa caldera for the last 1100 years, along with a classification of eruptive events and, with different colors (black for la Fossa cone and blue for Vulcanello), their relative source vent. The vertical arrows indicate the age of the eruptions, which produced deposits recognizable in the geological record, whereas their vertical length is proportional to the magnitude of the event. The two lines (continuous and dashed) represent the events for which the year is known from the historical record and those whose “best age” is placed taking into account the available age constraints, respectively. For the dated events, the chronological uncertainty is reported as the fork of the geochronological method. In the lower part of the figure, the inferred secondary-erosion and reworking-deposition scale occurred in response to tephra deposition is also reported. This diagram provides a clear indication that the activity of the two active centres within the La Fossa Caldera occurred in clusters of eruptions, with isolated events, such as the 1888–1890 eruption, being anticipated solely by phreatic events. These phreatic events can, therefore, be regarded as preparatory phenomena for the Vulcanian eruption. The diagram below illustrates the trend of sediment contribution (in the shaded portion of the gradient) from proximal areas, followed by a subsequent decrease (in the unshaded portion of the gradient) due to erosion, transportation, and re-sedimentation processes of volcaniclastic material

The time attribution of each tephra package is summarized in Table 1, whereas Fig. 13 graphically illustrates the overall eruptive history of La Fossa caldera for the last six centuries, along with a classification of eruptive events and their relative source vents (black color for la Fossa cone and blue for Vulcanello). The organization of Fig. 13 is conceived to offer the reader a synoptic picture of the primary and secondary processes that have characterized the history of the la Fossa caldera from 900 to 1900 AD. Compared to Di Traglia et al. (2013), the present work has, for the past four centuries, documented the eruptive history with greater precision by connecting eyewitnesses’ observations and tephra deposits.

A first observation concerns the number of volcanic eruptions: 30 in total, 25 from the La Fossa cone, located within the caldera, and 5 from Vulcanello, with an average of one eruption about every 33 yrs. The second major observation is the tendency of eruptions to occur in clusters separated by pauses of variable length. The length of quiescence ranges from a few years to over a hundred years, a pattern not uncommon at other volcanic systems (Selva et al. 2022). In this framework, it is also interesting that the present stasis is by far the longest in the past 1200 years, suggesting a high probability of a future reactivation.

With regard to eruptive styles, the most common explosive activity is represented by long-lasting, Vulcanian events. These are characterized by the repetitive Vulcanian bursts and by the deposition of tephra packages of dark-colored lapilli and ash. It is interesting of the ability of Vulcano to produce detonations heard from long distances, has been noted since medieval times, by the Arab geographer Abulfeda (1272–1331) who adopted the expression “island of thunder” and that of “island of lighting” for the activity of Vulcano and Stromboli, respectively, somehow anticipating the separation of the two styles of activity later introduced by Mercalli in the twentieth century.

The second most common explosive event (four cases) is phreatic eruptions (i.e., characterized by the emission of old, hydrothermally altered material). This style occurred mainly at the La Fossa cone and consisted of:

- the emission of variably energetic PDC ranging from impulsive, widely dispersed, blast-like currents poorly controlled by topography to diluted (surge-like) currents moderately controlled by topography to dense, fines-rich currents largely controlled by topography. The highly impulsive, blast-like events are by far the most hazardous phenomena due to a combination of PDC velocity and their association with heavy shower of ballistic blocks;
- repetitive, weak, gas and ash puffs. The eruptive clouds released by these events were vapor-rich and consisted of fine-grained, variably colored material as seen from the corresponding deposits. We suggest that a likely mechanism might be the mechanical milling of altered and fragile detritus clogging the bottom part of a funnel-shaped crater induced by periodic, energetic release of pressurized gas and vapor masses. The VT deposition likely had the double effect of blanketing the entire caldera with fine-grained, highly impermeable, cohesive ash beds (from Vulcanello to the Piano) and also of growth of the entire cone. Some VT beds exhibits presence of gravitational slumps, in agreement with the deposition from dry to variably wet eruptive clouds. The emission of lithic ash was, in some cases, gradually replaced by the emission of black-colored, juvenile material suggesting that the eruptions might have been controlled by gas bursts from a shallow magma column moving upwards causing a progressive shift, towards pure magmatic activity. The best example is provided by the topmost part of FF 1 (Fig. 6d).

The occurrence of the BdC-VT, by far the most energetic and violent eruption, was preceded by a period of quiescence. Rosi et al. (2018) suggested that the explosion resulted from a massive and rapid ascent of deep fluids promoted by the activation of a regional, N-S trending crustal fault. The inferred correlations between impulsive phreatic explosions and the activity of the NS-trending regional fault are predicated on three observations: (i) the synchronicity of the event with the Rocche Rosse event (Lipari), whose vent aligns with the same fault (Pistolesi et al., 2021); (ii) the alignment of the two phreatic events of the Forge craters, situated on the northern slope of the La Fossa cone, with the same tectonic line; (iii) the correspondence between the location of the source of Very Long Period (VLP) signals, indicating gas transfer from the hydrothermal system, and the fumarole areas within the caldera (La Fossa cone, Faraglione) during recent hydrothermal unrest (Di Traglia et al. 2023). The event ascribed to 1444 AD likely had the features of highly energetic lateral blast as the chronicles report blocks launched to distances of various km. Also in this case, this might fit with a mechanism that involved the rapid and massive rise of deep, pressurized fluids.

Other, less common eruptive styles are represented by sustained, transient, sub-Plinian (?) eruptions and by effusive phases.

The overall magmatic and volcanic evolution of eruptive activity can be conveniently referred to four major eruptive phases (Fig. 13): the first one, indicated in the diagram as Ancient Palizzi, occurred between 900 and 1050 AD and included PAL A, Vulcanello I and II and PAL B. This phase was likely controlled by a sustained, high-rate, injection of volatile-rich, hot mafic magma into the shallow magmatic system of the caldera (see Costa et al. 2023). The injection's effect led to an intense and prolonged Vulcanian cycle at the La Fossa cone (PAL A), coinciding with a significant and voluminous ascent of gas-poor magma at Vulcanello. This activity generated effusive eruptions and, to a lesser extent, Strombolian activity, which led to the construction of both the cone and the lava platform. Additionally, in conjunction with the activity responsible for forming the second cone, the effusion of gas-poor magma through a lateral, submarine vent, led to the emplacement of a submarine pillow lava field. The Ancient Palizzi activity ended with the eruption of PAL B, whose tephra directly overlies the lavas of Vulcanello platform I and also the tephra of Vulcanello cone II via the interposition of reworked sediments. The high energetic character of the eruption suggests that this magmatic phase was characterized by volatile-rich magma.

The second phase (Modern Palizzi) lasted approximately from 1050 to 1250 AD and included PAL C, PAL D and various lava flows (PAL E, PAL F, and PAL G). The activity was entirely concentrated at La Fossa cone and consisted of various Vulcanian eruptions, associated with lava flows, separated by short eruptive pauses. In addition, a sustained eruption ejecting trachyte pumice (PAL D) also occurred. PAL C was the most important Vulcanian event of this period and produced the most voluminous lava flow (Punte Nere). This was followed by the eruption of tephra (PAL D) which in turn preceded the emplacement of Commenda rhyolite lava flow, and the Campo Sportivo and Palizzi trachytic lava flows, each preceded by mild Vulcanian explosions (namely PAL E, PAL F, and PAL G, respectively). This effusive phase had likely the effect of filling the summit caters with a significant thickness of lava and also to produce a vertical growth of the cone.

After the phreatic eruptions of BdC and VT, a fresh injection of intermediate-mafic magma triggered renewed volcanic activity from 1400 AD to the mid-1500 s. Similar to the Ancient Palizzi eruptions occurred at both la Fossa and Vulcanello. Eruptions at La Fossa cone was only explosive (FF 1–5) and were characterized by both phreatic and Vulcanian events. The vents of phreatic activity have been identified both at the La Fossa cone (FF 1) and from two eccentric vents that formed on the northern flank of the cone, referred to as La Forgia Vecchia. Initially, FF 1 exhibited purely phreatic characteristics, ejecting hydrothermally altered ashes nearly indistinguishable from the underlying VT. Over time, this gave way to juvenile material, such as dark tephra, coarse dark bombs, and lava breccia, shifting from phreatic to Vulcanian activity. Additionally, new eruptions occurred at Vulcanello (Vulcanello IIIa/b), featuring tephra emissions during both phreatic and Vulcanian events, as well as lava emissions. During the fifteenth and sixteenth centuries, volcanic activity was absent but a few minor phreatic events, at both Vulcanello (IIIc/d) and La Fossa cone (PC 1).

From the beginning of eighteenth (PC 2–6) to the nineteenth centuries, volcanic activity ceased at Vulcanello, and resumed at La Fossa cone. After some significant phreatic events in 1731, significant, short-lived, moderate-lasting sustained eruptions of rhyolite pumice occurred in 1739 and 1771, followed by predominantly Vulcanian explosions. Only one effusive event, the Pietre Cotte obsidian rhyolite lava flow, occurred at la Fossa cone either at the end of 1771 eruption or in 1775.

The most recent eruption, occurred between 1888 and 1890, was a sort of isolated fairly large and long-lasting event which was preceded by a phreatic event in 1873. Further small explosions were reported in the historical record between the 1873 and the 1888, even if they did not leave recognizable deposits. Since 1890, La Fossa caldera has been quiet with volcanic activity marked only by intense hydrothermal activity and a strong fumarolic field at the top of la Fossa cone. Much less important fumaroles existed at the cone III of Vulcanello until the past century.

Only in a very few cases, low-angle, cross-bedded deposits observed either in natural outcrops or in trench’s cuts in deposits associated with long-lasting Vulcanian events (i.e., PAL A, PAL C1, C2, C3, FF 1, FF 3, FF 5, PC 6, GCb) were unequivocally recognized as compatible with dilute PDCs (Dellino et al. 2011 and references therein). We instead believe that by far the most common, high-angle, wavy-bedded, short-wavelength deposits, must be all attributed to processes of secondary remobilization. In their hazard assessment and review at Vulcano, Selva et al. (2020) emphasized that in the last 5,000 years, PDCs are rare and typically linked to Vulcanian activity. The study also noted uncertainties in the stratigraphic record and the absence of a thorough assessment of the completeness of the eruption history. The reconstruction presented in this study addresses these gaps by proposing a robust chrono-stratigraphic reconstruction and defining eruptive styles. This will enable a re-evaluation on the associated mean recurrence rates, particularly identifying a significantly higher frequency of Vulcanian and phreatic eruptions than previously recorded.

### The role of secondary remobilization processes at La Fossa caldera

Di Traglia et al. (2013) have already drawn attention to how the eruptive stasis and the shift of the vents between eruptive clusters are marked by unconformities and accompanied by modifications of the landscape surrounding the La Fossa cone. Moreover, some eruptions have shown inter-eruptive reworked deposits (e.g., the BdC and 1888–1890 eruptions; Di Traglia et al. 2013; Rosi et al. 2018).

**Fig. 14:**
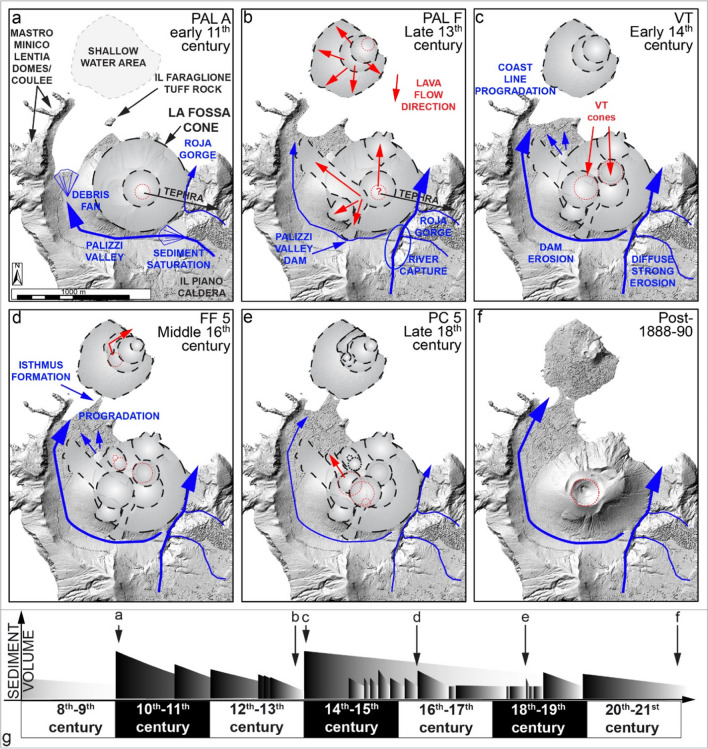
Landscape evolution of the La Fossa Caldera in the analyzed period. The large contribution of secondary volcaniclastic material occurs at the beginning of the considered period, with an impulse corresponding to PAL A, B, and C. The emplacement of lava flows, both at Vulcanello and La Fossa cone, has influenced morphological variations due to the low erodibility of the lavas, resulting in changes in surface outflow. The deposition of BdC and VT has caused significant changes in soil permeability, particularly on the La Fossa cone and its surrounding areas, especially in the E-SE direction. This increase in surface runoff and erosion has affected the more recent deposits, while preserving the older ones. Subsequently, Vulcanian activity has predominantly accumulated material in an E-SE direction, adding to the sediment supply through the Palizzi and Roja valleys during more voluminous eruptions (e.g., 1550, 1786, 1888–1890). Conversely, smaller eruptions did not cause significant morphological variations. During this period, two noteworthy morphogenetic phenomena include the two eccentric explosions in the Forgia Vecchia area and the emplacement of the Pietre Cotte lava flow

The volcanic history summarized above helps elucidate the interplay between: (i) secondary remobilization processes which accompanied and followed the emplacement of the main tephra units since the tenth century AD, and (ii) the complementary factors resulting from the growth of the la Fossa cone and the emplacement of lava flows. The effect of this interplay is illustrated in a sequence of six maps (Fig. 14) where each one is a tentative reconstruction of the geographic state of the caldera at the end of the different time windows. The blue lines and arrows indicate the flow directions of sediments, with the line thickness being qualitatively proportional to intensity of secondary processes. The main stages can be summarized as follows:

- Early-eleventh century AD: The La Fossa Caldera, breached to the NE in its submarine structure (Romagnoli et al. 2012; Casalbore et al. 2019), consisted of a marine gulf bounded to the W by a peninsula made by the ridge of the Mastro Minico-Lentia lava domes and coulées and to the south by the La Fossa cone. A small tuff rock (Il Faraglione) was possibly situated in the east sector of the bay. North of Il Faraglione, we hypothesize an area of shallow water, which extended northwards up to the area of the current Vulcanello for the presence of shallow seabed formed during the effusive activity of Roman times and continuously dismantled by marine erosion (Manni and Rosi 2021; Fig. 14a).

- 11th–thirteenth century AD: the onset of the Ancient Palizzi activity produced, in a short period, a massive accumulation of ash on the cone and east and south-east of La Fossa (Fig. 14b). The early mobilization paths of the tephra (main blue arrow) likely produced a massive sediment saturation within la Roja gorge followed by the formation of a debris fan in correspondence of the separation between the Palizzi drainage system and the La Roja gorge. The fan resulted in the diversion of the sediments coming from the Piano drainage system, towards the Palizzi (Di Traglia et al. 2013), likely producing a prograding delta on the coast at the valley terminal. In this same period, the most voluminous effusion of Vulcanello took place (Fusillo et al. 2015), which emplaced the lava shelf and the small composite cone (Vulcanello I and II cones), which together formed a small island on the northern margin of the La Fossa Caldera. Vulcanello likely emerged in an area where a shallow, flat seabed was formed as a result of deposition and subsequent erosion by sea abrasion of eruptive material associated with the eruptions described during the Classical period (second-first century BC; Manni and Rosi 2021). This area is inferred from: (i) the absence of clear lava-sea interaction structures, and (ii) the horizontally oriented basal surfaces of the different lava flows that constitute the lava field.
- Late thirteenth century AD: the landscape scenario further evolved during Modern Palizzi phase as the sediment supply to the coastline continued and the shore steadily advanced northwards. Shortly later, four lava flows were emplaced: Punte Nere (AD 1000–1170) on the N-NE slope, Commenda (AD 1150–1350) on the E-SE slope, Campo Sportivo (undated), Palizzi (AD 1240–1300) on the NW and S-SE slopes, respectively. It is interesting that the Campo Sportivo lava shows no signs of interaction with water (pseudo-pillows or hyaloclastic zones), suggesting that the coastline was already further north. The emplacement of the lava flow also stopped sediments spreading eastwards and likely forced transport in the narrow sector between the lava and the Lentia Ridge. The Palizzi lava also constricted the Palizzi valley and induced erosion on the opposite side of the valley. A further effect of damming was produced by the accumulation of BdC PDC deposits (Fig. 5). This was followed by an aggressive erosional phase which dissected older deposits and displaced a large mass of BdC and pre-Ancient Palizzi tephra deposits downstream.
- Early fourteenth century AD: following the eruption of BdC and the VT ash emission deposition, the La Fossa cone continued to grow (Fig. 14c) whereas the entire watersheds of the island became sealed with very fine-grained, cohesive ash, with abundant clay minerals of hydrothermal origin (Capaccioni and Coniglio 1995). VT had hydraulic characteristics of making the surface impermeable and resistant to erosion, with a following catastrophic increased runoff, exacerbating erosion and transport downstream towards the Porto plain volcano of both fine ash and ancient deposits (Bonasia et al. 2022). During this phase, the coastline and in particular that at the foot of the Lentia ridge rapidly migrated northwards.
- Middle sixteenth century AD: after a temporary reduction in the second half of fifteenth century, sediment transport in the Palizzi valley resumed, promoted by new Vulcanian activity (Fig. 14d). This caused a substantial accumulation of material between Lentia and Campo Sportivo lava flow whereas the northern shore migrated northwards until an initial isthmus formed between Vulcano and Vulcanello. In this period, a valley formed between the Forgia Vecchia and the lava flow of the Campo Sportivo, which is plausibly the valley from which De Luc climbed to the top in 1757 and into which the Pietre Cotte lava subsequently flowed.
- Late eighteenth century AD and post-1888–1890: the blue arrow of the Palizzi valley indicate a continuous supply of sediment that resulted in a further rise of the ground level in the sector of the caldera east of Lentia ridge and also a further growth and expansion of the isthmus (Fig. 14 e, f).

The reconstruction of the evolution of La Fossa caldera highlights the key role of primary and secondary processes in shaping the bottom part of the caldera, where an impressive accumulation of secondary volcaniclastic deposits resulted in rapid and dramatic changes in the geography of the caldera. Since the fourteenth century, the western sector underwent a more pronounced accumulation of sediment as a result of the confinement exerted by the caldera walls and the new lava tongue of Campo Sportivo. Although the thickness of secondary deposits accumulated within the caldera is a key information yet difficult to obtain, artificial excavations carried out during the periods of the survey sporadically made available a more completed stratigraphy of the caldera bottom. Among the various excavations, the very large and up to 7 m depth, digging made for the construction of the desalination plant resulted to be particularly useful. As illustrated in Fig. 11, the accumulation of secondary volcaniclastic material progressed from the second half of eighteenth to the end of nineteenth centuries in the western sector. It is interesting noticing that the area has been characterized by a ground level rise ranging from 2 (Fig. 11c) to 7 m (Fig. 11b). Extrapolating this trend to the future suggests that the area would be severely exposed to ground rise (probably in the order of meters) in case of volcanic activity resumption. The identification of post-eruptive phenomena and the estimation of associated hazards are central themes in volcanology, as evidenced by numerous studies (Pierson and Major 2014; Major et al. 2019; Di Traglia 2020; Di Vito et al. 2023). However, this topic is often underestimated within active calderas due to the scarcity of natural outcrops within them. The morphology of calderas often leads to the accumulation of thick deposits, masking stratigraphic sequences. This poses a significant challenge for hazard evaluation studies within calderas, and therefore, it can no longer be overlooked.

## Conclusions

Using the case study of La Fossa caldera (Vulcano island, Italy), we have presented a comprehensive approach that can facilitate the characterization of volcanic systems with low-to-high intensity activity intercalated with significant erosional phases. Our analyses document with unprecedented accuracy the chrono-stratigraphy of La Fossa caldera where eruptions of relatively small volume and moderate intensity, which originated from multiple vents, have complicated the reconstruction of the eruptive history. Many different information sources and observations have been integrated to unravel aspects of the volcanic system that would be otherwise impossible to be deduced. This has provided fundamental insights into hazard identification and characterization. In particular, extensive and ad-hoc planned field studies (including natural sections as well as trenches dug in strategic sites) were combined with a vast set of chronological constraints (^14^C and paleomagnetism dating) and a new analysis of historic chronicles. We documented a total of 30 major eruptive events captured in the stratigraphic record, 25 of which were associated with La Fossa volcano and 5 with the Vulcanello cones, including 4 phreatic events and 7 effusive eruptions. The average frequency was one eruption every 35 years, but most eruptions are concentrated in clusters and only a few occurred as isolated events (e.g., BdC around 1300, 1636, and 1888–1890, although the latter was preceded by a series of phreatic explosions, the most energetic of which occurred in 1873). We also conclude that the hydrothermal system played an important role in the explosivity as its presence likely represented a pre-requisite element necessary to allow the occurrence of impulsive, highly energetic blasts (BdC and La Forgia 1444 events), probably linked to the tectonic activity of the crustal, N-S trending fault connecting Lipari to Vulcano. An important feature that the work contributes to unravel is the role of milling fragmentation of hydrothermally altered material during periods in which gas puffing was sustained by the rise of an active magma column within a conduit clogged upwards with hydrothermally altered debris. Given that the current repose period is the longest in the past 1100 years, the probability of future reactivation is to be considered high.

Another important achievement of this work was to highlight the hazard posed by long-lasting, secondary mobilization of loose volcaniclastic material following explosive eruptions. This phenomenon is exacerbated by the presence of the caldera depression and controlled by a complex interplay between tephra deposition on steep slopes (mostly ash), vent migration, emplacement of lava flows, and repeated diversion of the drainage network induced by sediment saturation. The potential burial of buildings and infrastructure by debris flows is significant in case of volcano reactivation, particularly in the sector of the caldera at the foot of the Lentia ridge, due to the sediment supply along the Palizzi valley. Over the past three centuries, the average ground increase has been of more than 2 m/century and such a rate was possibly even higher in the previous centuries. Unfortunately, the lack of knowledge of this hazard combined with the absence of integration of hazard aspects in urban planning has contributed to the development of Porto town and to the construction of critical infrastructure (power, desalination, and sewage treatment plants), almost all located in the sector with the highest probability of burial from debris flows and floods.

This study finally demonstrates the need for accurate characterization of past activity in volcanic systems, whether they exhibit low or high intensity. This characterization relies on a combination of multiple aspects, such as stratigraphic correlations, tephra mapping, historical chronicles, and geochronology. The aim is to effectively identify potential hazards and develop dedicated strategies for risk management and reduction.
